# Acute, chronic and conditioned effects of intranasal oxytocin in the mu-opioid receptor knockout mouse model of autism: Social context matters

**DOI:** 10.1038/s41386-024-01915-1

**Published:** 2024-07-17

**Authors:** Fani Pantouli, Camille N Pujol, Cécile Derieux, Mathieu Fonteneau, Lucie P. Pellissier, Claire Marsol, Julie Karpenko, Dominique Bonnet, Marcel Hibert, Alexis Bailey, Julie Le Merrer, Jerome A. J. Becker

**Affiliations:** 1 INRAE, CNRS, Université de Tours, Inserm, PRC, 37380 Nouzilly, France; 2 Florida Research & Innovation Center, Cleveland Clinic, 9801 SW Discovery Way, Port St. Lucie, FL 34987 USA; 3 Pharmacology section, Institute of Medical and Biomedical Education, St George’s University of London, London, SW17 ORE UK; 4 Department of Psychiatry, Strasbourg University Hospital, 67091 Strasbourg, France; 5 UMR1253, iBrain, Université de Tours, Inserm, CNRS, Faculté des Sciences et Techniques, Parc de Grandmont, 37200 Tours, France; 6 Laboratoire d’Innovation Thérapeutique, Faculté de Pharmacie, UMR7200 CNRS/Université de Strasbourg, 74 route du Rhin, 67412 Illkirch, France

**Keywords:** Autism spectrum disorders, Social neuroscience

## Abstract

Autism Spectrum Disorders (ASD) are neurodevelopmental disorders whose diagnosis relies on deficient social interaction and communication together with repetitive behaviours. Multiple studies have highlighted the potential of oxytocin (OT) to ameliorate behavioural abnormalities in animal models and subjects with ASD. Clinical trials, however, yielded disappointing results. Our study aimed at assessing the behavioural effects of different regimens of OT administration in the *Oprm1* null mouse model of ASD. We assessed the effects of intranasal OT injected once at different doses (0.15, 0.3, and 0.6 IU) and time points (5, 15, and 30 min) following administration, or chronically, on ASD-related behaviours (social interaction and preference, stereotypies, anxiety, nociception) in *Oprm1*^*+/+*^ *and Oprm1*^*-/-*^ mice. We then tested whether pairing intranasal OT injection with social experience would influence its outcome on ASD-like symptoms, and measured gene expression in the reward/social circuit. Acute intranasal OT at 0.3 IU improved social behaviour in *Oprm1*^*-/-*^ mice 5 min after administration, with limited effects on non-social behaviours. Chronic (8–17 days) OT maintained rescuing effects in *Oprm1* null mice but was deleterious in wild-type mice. Finally, improvements in the social behaviour of *Oprm1*^*-/-*^ mice were greater and longer lasting when OT was administered in a social context. Under these conditions, the expression of OT and vasopressin receptor genes, as well as marker genes of striatal projection neurons, was suppressed. We detected no sex difference in OT effects. Our results highlight the importance of considering dosage and social context when evaluating the effects of OT treatment in ASD.

## Introduction

Autism Spectrum Disorders (ASD) are highly heritable neurodevelopmental disorders characterized by impaired social communication and interaction associated with a restricted, repetitive repertoire of behaviours, interests, and activities [1]. Alongside these core symptoms, ASD is frequently associated with comorbid symptoms such as high anxiety, cognitive impairment, motor stereotypy, aggressive behaviour, abnormalities in pain sensitivity, and epilepsy [2, 3]. Despite the identification of vulnerability genes and environmental risk factors [4, 5], the etiopathological mechanisms underlying ASD remain essentially unknown. To date, approved pharmacological treatments for ASD mostly target associated symptoms [6, 7] and evidence-based behavioural interventions remain the only treatments proven to ameliorate core social deficits [8].

Among potential pharmacological treatments for ASD, oxytocin (OT) stands out as a highly promising molecule to relieve socio-communicational impairments. OT is a neuropeptide synthesized in the paraventricular (PVN) and supraoptic nuclei (SON) of the hypothalamus. Animal research has evidenced, beyond its key contribution to reproductive functions, a crucial role for this nonapeptide in shaping social behaviours, including social approach and reward [9, 10], social recognition [11] and memory [12], parental behaviour [13], pair bonding [14] and emotion discrimination [15]. Consistent with this, targeted disruption of genes encoding OT and its receptor (OTR) impairs social behaviour in mice [16, 17]. Experiments in mice have shown that OT is released in response to social cues, making it a key contributor to the rewarding properties of social interaction [6, 9]. In human studies, OT effects have been examined after intranasal administration as oral intake does not allow for sufficient bioavailability [18, 19]. In healthy subjects, intranasal OT increases social salience [20, 21], improves facial emotional recognition [22], and promotes in-group cooperation and trust [23, 24].

Prosocial effects under physiological conditions bode well for the therapeutic effects of exogenously administered OT in ASD. Preclinical studies in mouse models have evidenced improvements in social interaction [25, 26], social preference [27–30], or social memory [31, 32] under OT treatment. Such prosocial effects persisted upon (sub)chronic administration or when OT was administered early in life [26, 27, 30]. Accordingly, in patients with ASD, acute intranasal OT application improved sustained eye gaze and social cooperation, but had limited effects on non-social behaviours [33, 34]. Unfortunately, clinical trials testing long-term, daily OT exposure in ASD yielded inconsistent results, with the largest to-date clinical trial showing no effect of intranasal OT on social behaviours in children with the condition [35, 36].

Several factors may have contributed to these disappointing outcomes. Most clinical trials omitted to consider the heterogeneity of ASD, as chronic OT could be beneficial in a small subset of individuals [37, 38] and to test multiple or individualized doses [36, 39]. Moreover, animal studies suggest that the regimen of OT treatment needs to be considered, as chronic administration leads to severe social deficit in wild-type mice [40]. Finally, OT does not always behave as a facilitator of social behaviour [21, 41] and its prosocial effects would depend on social context [42], as predicted by the social salience hypothesis [21]. Taking all the above into consideration, it appears that chronic intranasal OT treatment, at a dose and social context not individually adapted, may not be optimal as an ASD therapeutic approach [36, 43, 44].

In this study, we challenged previous hypotheses by assessing the behavioural consequences of varying the dose, timing, and context of intranasal OT administration in the mu-opioid receptor knockout (*Oprm1*^*-/-*^) mouse model of ASD. The *Oprm1*^*-/-*^ model is a well characterised mouse model of ASD mouse [45–48] with an altered oxytocinergic system [45, 46, 49, 50]. Upon acute administration of OT or a non-peptide analogue, these mice show rescued communication and social interaction [50, 51]. Here, we evaluated the effect of a range of doses of single intranasal OT administration on social behaviour in *Oprm1*^*+/+*^ and *Oprm1*^*-/-*^ mice and assessed the contribution of OTR in mediating these effects using a novel OT antagonist. We also tested whether acute OT would affect other, non-social, autism-sensitive behaviours. We then evaluated the effects of intranasal OT administration in a chronic setting. Finally, we assessed the behavioural consequences of pairing intranasal OT injection with congener versus object presentation in *Oprm1*^*-/-*^ mice and *Oprm1*^*+/+*^ controls. To gain insight into the molecular substrate of OT effects, we measured gene expression, notably for genes related to the oxytocin/vasopressin system, in several regions of the reward/social circuit.

## Methods

### Ethics

This study was approved by the Comité d’Ethique pour l’Expérimentation Animale de l’ICS et de l’IGBMC (Com’Eth, 2012-033) and Comité d’Ethique en Expérimentation animale Val de Loire (C2EA-19). All experimental procedures were conducted in accordance with the European Communities Council Directive 2010/63/EU. Animal studies are reported in compliance with the ARRIVE guidelines [52] and with the recommendations made by the British Journal of Pharmacology [53].

### Animals

Equivalent numbers of male (25–32 g) and female (22–28 g) *Oprm1*^*+/+*^ and *Oprm1*^*-/-*^ mice [54] were bred in-house on an identical hybrid background: 50% 129SVPas - 50% C57BL/6J. We defined sample size (GPower 3.1) to ensure enough statistical power using ANOVA or Kruskal-Wallis analysis of variance to detect significant effect on our parameters (effect size f = 1.80, α = 0.05, σ = 5, n = 8, power = 0.96). *Oprm1*^*+/+*^ and *Oprm1*^*-/-*^ mice were bred from homozygous parents, which were bred from heterozygous animals, to prevent genetic derivation; mice in the same cage were of the same genotype. This breeding scheme and housing conditions likely favoured social deficits in mutant mice by maintaining them together during early post-natal development [45, 49]. The size of litter was not standardized (dams were housed in pairs). Mice were weaned at 3-week age. Cages containing *Oprm1*^*+/+*^ or *Oprm1*^*-/-*^ mice (same age and sex) were organised from as many different litters as possible (to limit litter effect) by the staff of the animal facility (blind to experiments) and assigned randomly to a treatment condition (same treatment in the whole cage). We ensured that sex ratio was equivalent between groups, and that mice from different litters met during the direct social interaction test. Except for a 30-min isolation before the novelty-suppressed feeding test, mice were all maintained group-housed (2–4 mice per cage), on a 12 hr light/dark cycle (lights on at 7:00 AM) at controlled temperature (21 ± 1 °C). Food (except before the novelty-feeding test) and water were available *ad libitum*.

### Drugs and treatments

Mice received either vehicle (NaCl 0.9%) or oxytocin (PubChem ID: 439302; reference #03251, Sigma Aldrich, Saint Quentin, France) at the dose of 0.15 IU (∼400 µg/kg), 0.3 IU (∼800 µg/kg) or 0.6 IU (∼1600 µg/kg) via the nasal route, either acutely (5, 15 or 30 min before testing), chronically (once a day for 17 consecutive days, 5 min before testing) or repeatedly (6 administrations, 2–3 days apart, 5 min before testing). A solution of 1 IU contained 1.667 mg of synthetic OT. For intranasal OT administration, we adapted our protocol from [40]. OT was dissolved in saline vehicle (0.9% NaCl) and administered in both nostrils in a volume of 5 μl to each mouse. OT doses used in this study were similar to high doses of intranasal OT given to adolescent prairie voles [55] and in the lower range of the daily dosage used in clinical trials [35, 36, 56]. To assess whether the OT effects were mediated by OTR activation, we administered via intraperitoneal route either vehicle (carboxymethyl cellulose 1% in NaCl 0.9%) or the highly selective OT receptor antagonist LI183, synthesized in-house, at the dose of 7.5 or 15 mg/kg (synthesis and pharmacological profile in Supplement 1) 30 min before behavioural testing.

### Behavioural experiments

**Fig. 1:**
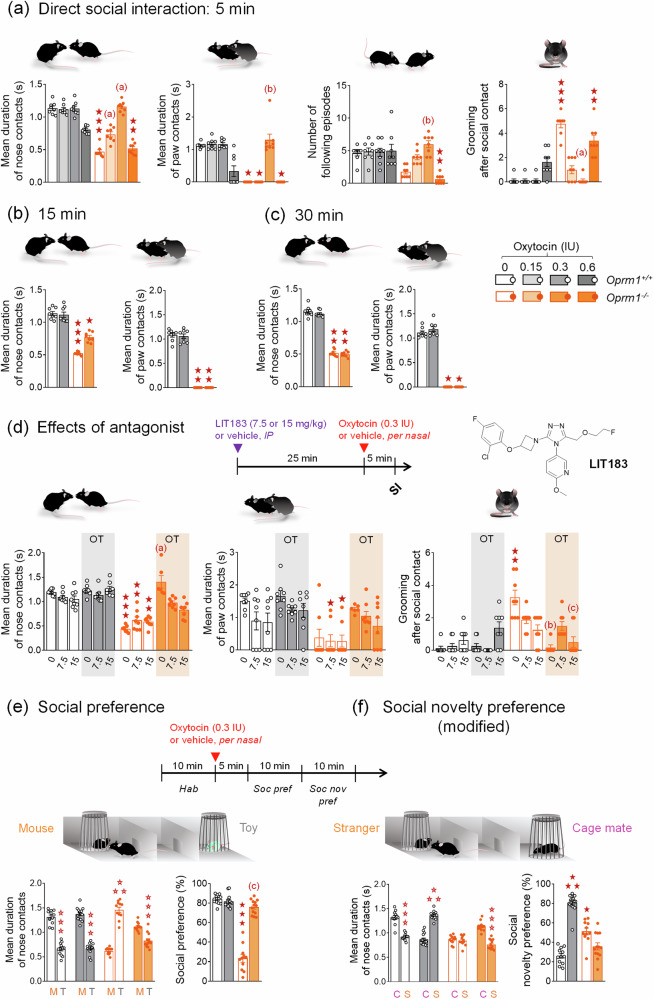
Acute per nasal administration of OT dose-dependently restored social behaviour in *Oprm1* null mice. **a** *Oprm1*^*+/+*^ and *Oprm1*^*-/-*^ mice received OT or vehicle (4 males – 4 females per genotype and treatment), via per nasal route, 5 min before the direct social interaction test and at the dose of 0, 0.15, 0.3, or 0.6 IU. In this test, vehicle-treated *Oprm1*^*-/-*^ mice displayed a deficit in social interaction; this deficit was partially reversed for the OT dose of 0.15 IU, fully relieved at 0.3 IU, and remained unchanged at 0.6 IU (mean duration of nose contacts: *H*_*7,64*_ = 53.13*, p* < 0.0001, mean duration of paw contacts: *H*_*7,64*_ = 49.93*, p* < 0.0001, number of following episodes: *H*_*7,64*_ = *37.57, p* < 0.0001, grooming after social contact: *H*_*7,64*_ = 49.76*, p* < 0.0001). **b** When administered 15 min before testing (4 males – 4 females per genotype and treatment), the optimal dose of 0.3 IU had only partial effects on the duration of nose contacts (*H*_*3,32*_ = 26.28*, p* < 0.0001*)* in *Oprm1*^*-/-*^ mice and no effect on the duration of paw contacts (*H*_*3,32*_ = 26.59*, p* < 0.0001). **c** When administered 30 min before testing (4 males – 4 females per genotype and treatment), per nasal OT at 0.3 IU was ineffective in relieving social interaction deficit in *Oprm1* null mice. **d** The non-peptide OT antagonist LIT183 (see Supplement 1) or its vehicle (doses of 0, 7.5, or 15 mg/kg) were administered intraperitoneally 25 min before per nasal OT administration (0.3 IU) and 30 min before direct social interaction test (4 males – 4 females per genotype, LIT183 doses and OT treatment). In *Oprm1*^*-/-*^ mice, LIT183 blunted the effects of intranasal OT on the mean duration of nose (*H*_*11,94*_ = 74.35*, p* < 0.0001) but not paw contacts (*H*_*11,94*_ = *74.35, p* < 0.0001) and reduced grooming after social contact at 7.5 mg/kg, (*H*_*11,94*_ = 57.01*, p* < 0.0001); OT antagonist reduced such grooming in mutant mice treated with vehicle. **e** We performed a modified version of the 3-chamber test (*Oprm1*^*+/+*^ vehicle: 6 males – 7 females, *Oprm1*^*+/+*^ OT: 7 males - 8 females, *Oprm1*^*-/-*^ vehicle: 5 males - 8 females, *Oprm1*^*-/-*^ OT: 6 males – 8 females). During the social preference phase, intranasal OT restored preference for making longer nose contacts with the mouse versus the object in *Oprm1* null mice (*Genotype x Dose x Stimulus: F*_*1,47*_ = 124.01*, p* < 0.0001), resulting in a fully rescued social preference ratio (*H*_*3,51*_ = 30.35*, p* < 0.0001). No effect was detected in *Oprm1*^*+/+*^ mice. **f** During the modified social novelty preference phase, OT triggered a preference for making longer nose contacts with the cage mate versus the novel mouse in *Oprm1*^*-/-*^ mice; in contrast, this treatment shifted the preference of *Oprm1*^*+/+*^ mice towards making longer nose contacts with the novel mouse (*G x D x S: F*_*1,47*_ = 136.62*, p* < 0.0001). Similar opposite effects were detected for the social novelty preference ratio (*H*_*3,51*_ = 37.15*, p* < 0.0001). Results are shown as scatter plots and mean ± sem. Solid stars: significant difference with the vehicle-treated *Oprm1*^*+/+*^ group, Tuckey’s post-hoc test following a two-way ANOVA or 2-tailed t-test following a Kruskal-Wallis analysis of variance; open stars: genotype x treatment x stimulus interaction (stimulus: mouse/toy or stranger/cage mate comparison), Tukey’s post-hoc test following a repeated measure analysis of variance (ANOVA); one symbol: *p* < 0.05, two symbols: *p* < 0.01; three symbols: *p* < 0.001. Letters: significant difference with vehicle-treated *Oprm1*^*-/-*^ group (2-tailed t-test or Tukey’s post-hoc test); (**c**): *p* < 0.05, (b): *p* < 0.01, (**a**): *p* < 0.001. More behavioural parameters in Fig. S2. C cage mate, IU International Units, M mouse, OT oxytocin, S stranger, SI social interaction, T toy.

**Fig. 2:**
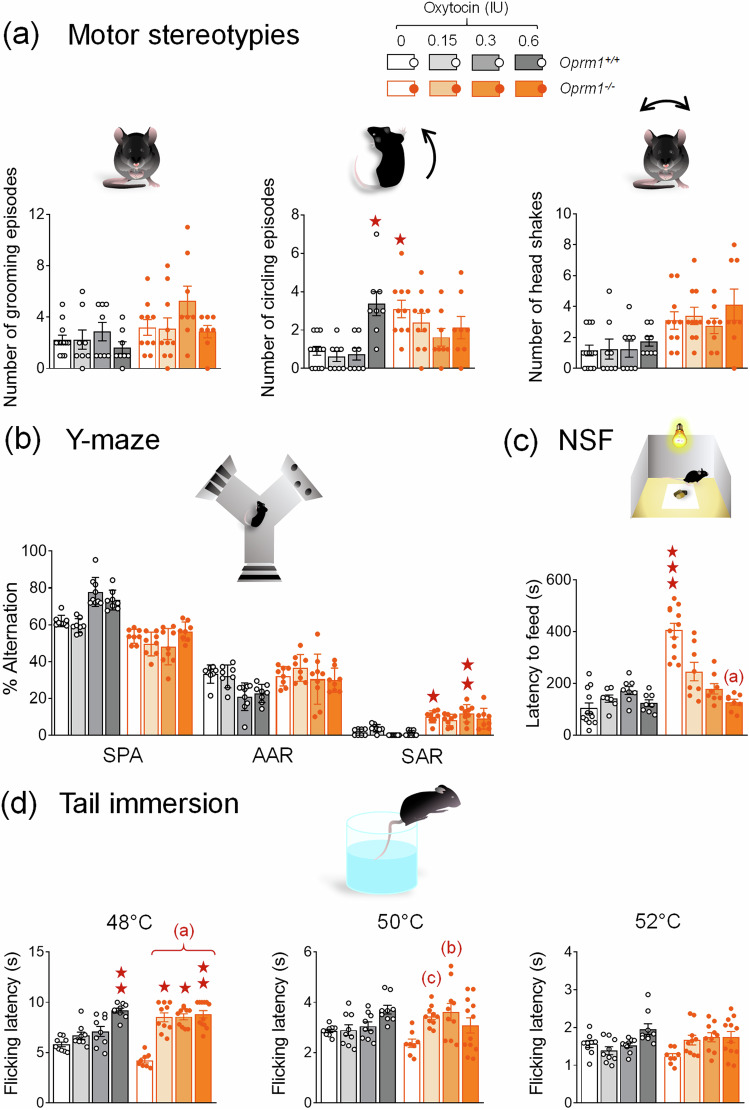
Acute per nasal OT relieved anxiety and induced analgesic effects in *Oprm1* null mice but had limited effects on stereotypies and perseveration. **a** When administered acutely 5 min before monitoring spontaneous motor stereotypies (*Oprm1*^*+/+*^ vehicle: 6 males – 6 females, *Oprm1*^*-/-*^ vehicle: 5 males - 5 females, *Oprm1*^*-/-*^ OT 0.15 IU: 4 males – 6 females, other groups: 4 males – 4 females per genotype and dose), per nasal OT increased the number of circling events in *Oprm1*^*+/+*^ mice. Vehicle-treated *Oprm1* null mice displayed more frequent circling behaviour, reduced under OT administration (*H*_*7,72*_ = *29.78, p* < 0.0001). **b** When exploring the Y-maze (*Oprm1*^*-/-*^ OT 0.3 and 0.6 IU: 4 males – 5 females, other groups: 4 males – 4 females per genotype and dose), vehicle-treated and 0.3 IU OT-treated *Oprm1*^*-/-*^ mice exhibited more frequent same arm returns than *Oprm1*^*+/+*^ control mice *(H*_*7,66*_ = *24.1, p* < 0.01); this perseverative behaviour was not detected in 0.15 and 0.6 OT-treated mutant mice. **c** In the novelty-suppressed-feeding test (*Oprm1*^*+/+*^ and *Oprm1*^*-/-*^ vehicle^*:*^ 6 males – 6 females, other groups: 4 males – 4 females per genotype and dose), increased latency to feed in *Oprm1*^*-/-*^ mice was normalized under OT administration (*H*_*7,72*_ = *39,8, p* < 0.0001). **d** In the tail immersion test (*Oprm1*^*+/+*^ groups: 4 males – 5 females, *Oprm1*^*-/-*^ vehicle: 4 males – 4 females; *Oprm1*^*-/-*^ OT at 0.15 and 0.5 IU: 5 males ^*–*^ 5 females, *Oprm1*^*-/-*^ OT at 0.6 IU: 5 males – 7 females), OT-treated mice (0.6 IU in wild-type mice, all doses in *Oprm1* null mice) showed analgesia compared to saline-treated *Oprm1*^*+/+*^ mice at 48 °C (*H*_*7,76*_ = *49.4, p* < 0.0001). At 50 °C, 0.15 and 0.3 IU of OT produced analgesic effects in *Oprm1*^*-/-*^ mice (*H*_*7,76*_ = *21.4, p* < 0.01). At 52 °C, OT increased nociceptive thresholds only in *Oprm1*^*-/-*^ mice, at doses of 0.15 and 0.3 IU. No significant effect of OT was detected. Results are shown as scatter plots and mean ± sem. Solid stars: significant difference with the vehicle-treated *Oprm1*^*+/+*^ group, Tuckey’s post-hoc test following a two-way ANOVA or 2-tailed t-test following a Kruskal-Wallis analysis of variance. Letters: significant difference with vehicle-treated *Oprm1*^*-/-*^ group (2-tailed t-test); (**c**): *p* < 0.05, (**a**): *p* < 0.001. AAR alternate arm returns, NSF novelty-suppressed feeding, SAR same arm returns, SPA spontaneous alternation.

**Fig. 3:**
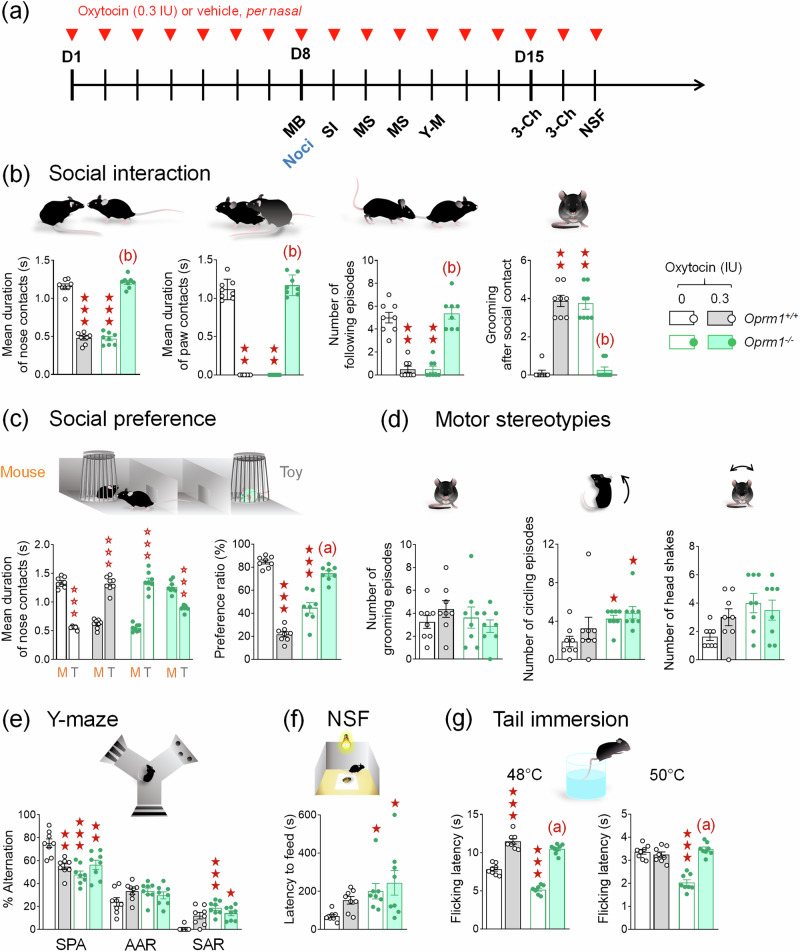
Chronic intranasal OT maintained prosocial effects in *Oprm1* knockout mice while producing a severe social deficit in wild-type controls. **a** A first cohort of *Oprm1*^*+/+*^ and *Oprm1*^*-/-*^ mice was treated daily with either OT (0.3 IU) or vehicle (4 males – 4 females per genotype and treatment) via per nasal route for 17 days. Behavioural testing started on D8. A second cohort received OT (0.3 IU) or vehicle (4 males – 4 females per genotype and treatment) daily for 8 days and was tested for nociception on D8 (blue characters). **b** In the direct social interaction test, chronic OT restored interaction parameters in *Oprm1*^*-/-*^ mice while it resulted in a severe deficit in *Oprm1*^*+/+*^ mice (mean duration of nose contacts: *G x T: F*_*1,28*_ = 666.2*, p* < 0.0001; mean duration of paw contacts: *H*_*3,32*_ = 26.8*, p* < 0.0001, number of following episodes: *H*_*3,32*_ = 24.3*, p* < 0.0001, grooming after social contact: *H*_*3,32*_ = *25.5, p* < 0.0001). **c** Similarly, in the social preference test, repeated OT exposure compromised preference for the mouse over the toy in *Oprm1*^*+/+*^ mice, but rescued this preference in *Oprm1*^*-/-*^ mice (mean duration of nose contacts: *Genotype x Treatment x Stimulus: F*_*1,28*_ = 789.8*, p* < 0.0001, preference ratio: *G x T: F*_*1,28*_ = 252.1*, p* < 0.0001). **d** *Oprm1*^*-/-*^ mice display more frequent circling behaviour, and OT administration had no influence on this stereotyped behaviour (*H*_*3,32*_ = 13.2*, p* < 0.01); no effect was detected in *Oprm1*^*+/+*^ controls. **e** In the Y-maze, chronic OT failed to suppress perseverative same arm entries (SAR) in *Oprm1* mutants, and impaired spontaneous alternation (SPA) in *Oprm1*^*+/+*^ mice (*G x T: F*_*1,28*_ = 17.8*, p* < 0.001*)*. **f** In the novelty-suppressed feeding test, OT failed to relieve increased latency to eat in *Oprm1*^*-/-*^ mice (*H*_*3,32*_ = *13.1, p* < 0.01). **g** In the tail immersion test, OT normalized nociceptive thresholds in *Oprm1*^*-/-*^ mice at 48 °C, while inducing analgesia in *Oprm1*^*+/+*^ controls (*G x T: F*_*1,28*_ = 10.3*, p* < 0.01). At 50^*°*^C, chronic OT normalised nociceptive thresholds in *Oprm1*^*-/-*^ mice, without effects in WT mice (*G x T: F*_*1,28*_ = 50.2*, p* < 0.0001). Results are shown as scatter plots and mean^ *±* ^sem. Solid stars: significant difference with the vehicle-treated *Oprm1*^*+/+*^ group, Tuckey’s post-hoc test following a two-way ANOVA or 2-tailed t-test following a Kruskal-Wallis analysis of variance; open stars: genotype x treatment (Y-maze) or genotype x treatment x stimulus interaction (Social preference - stimulus: mouse/toy), Tukey’s post-hoc test following an analysis of variance (ANOVA); one symbol: p < 0.05, two symbols: *p* < 0.01; three symbols: *p* < 0.001. Letters: significant difference with vehicle-treated *Oprm1*^*-/-*^ group (2-tailed t-test or Tukey’s post-hoc test); (**c**): *p* < 0.05, (**b**): *p* < 0.01, (**a**): *p* < 0.001. More behavioural parameters in Fig. S3. 3-Ch: 3-chamber social preference test, AAR alternate arm returns, D day, MB marble burying, MS motor stereotypies, Noci nociception, NSF novelty-suppressed feeding, SAR same arm returns, SPA spontaneous alternation, Y-M Y-maze.

**Fig. 4:**
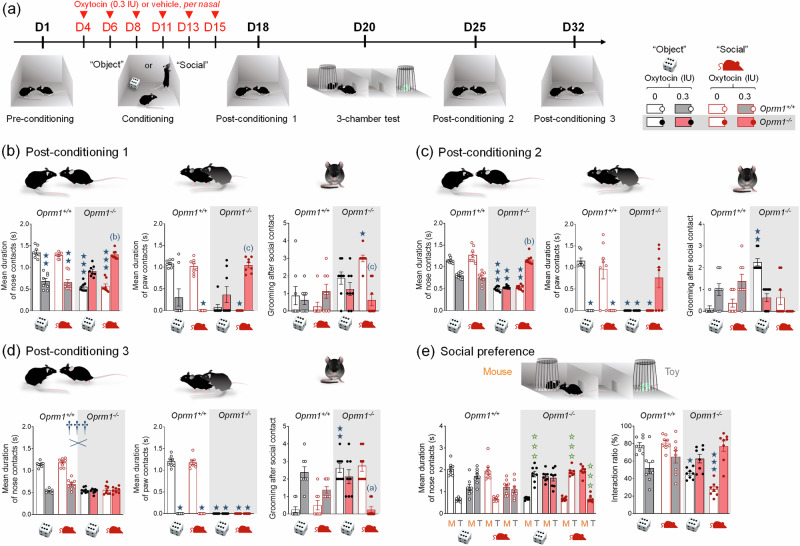
Prosocial effects of repeated intranasal OT on social deficit in *Oprm1* null mice were greater and lasted longer when associated with social experience. **a** After a pre-conditioning social interaction session, mice received per nasal OT (0.3 IU) or vehicle administration paired with the presentation of an unfamiliar object (“object” condition) or mouse (“social” condition) every two/three days over 2 weeks (D4 to D15) (4 males – 4 females per genotype, treatment, and conditioning paradigm). A first post-conditioning social interaction session took place on D18, two days before 3-chamber test for social novelty preference (D20). Social interaction was assessed during two additional post-conditioning sessions, a week (D25) and two weeks (D32) after the first post-conditioning session. **b** During the first post-conditioning social interaction session, OT-treated *Oprm1*^*+/+*^ mice displayed significant deficits in social behaviour. In contrast, OT improved social behaviour in *Oprm1*^*-/-*^ mice (mean duration of nose contacts: *H*_*7,64*_ = 50.5*, p* < 0.0001), more efficiently in mice tested under the “social” paradigm (mean duration of paw contacts: *H*_*7,64*_ = *37.4, p* < 0.0001; grooming after social contact: *H*_*7,64*_ = *27.3, p* < 0.001). **c** After a week, impaired social interaction was still detected in *Oprm1*^*+/+*^ mice; among OT-treated *Oprm1*^*-/-*^ mice, only those tested under the “social” paradigm displayed a restoration of social behaviour (mean duration of nose contacts: *H*_*7,64*_ = *54.6, p* < 0.0001*;* mean duration of paw contacts: *H*_*7,64*_ = *44.9, p* < 0.0001; grooming after social contact: *H*_*7,64*_ = 36.06*, p* < 0.0001). **d** After another week, while a social behaviour deficit was still observed in OT-treated *Oprm1*^*+/+*^ mice, some prosocial effects of OT conditioning were maintained for *Oprm1*^*-/-*^ mice when tested under the social paradigm only (mean duration of nose contacts: *Genotype x Treatment: F*_*1,56*_ = *189,3, p* < 0.0001*;* mean duration of paw contacts: *H*_*7,64*_ = 61.3*, p* < 0.0001; grooming after social contact: *H*_*7,64*_ = 44.7*, p* < 0.0001*)*. **e** In the three-chamber test, we observed a full restoration of social preference when *Oprm1*^*-/-*^ mice were exposed to OT under the “social” but not “object” setting (mean duration of nose contacts: *Stimulus x Treatment x Paradigm: F*_*1,28*_ = 27.8, *p* < 0.0001; preference ratio: *H*_*7,64*_ = *38.0, p* < 0.0001). Results are shown as scatter plots and mean ± sem. Solid stars: significant difference with the vehicle-treated *Oprm1*^*+/+*^ group, Tuckey’s post-hoc test following a two-way ANOVA or 2-tailed t-test following a Kruskal-Wallis analysis of variance; open stars: genotype x treatment (Y-maze) or genotype x treatment x stimulus interaction (Social preference - stimulus: mouse/toy or stranger/cage mate comparison), Tukey’s post-hoc test following an analysis of variance (ANOVA); daggers: genotype x treatment interaction; one symbol: *p* < 0.05, two symbols: *p* < 0.01; three symbols: *p* < 0.001. Letters: significant difference with vehicle-treated *Oprm1*^*-/-*^ group (2-tailed t-test or Tukey’s post-hoc test); (**c**): *p* < 0.05, (**b**): *p* < 0.01, (**a**): *p* < 0.001. More behavioural parameters in Fig. S4. D day, M mouse, T toy.

**Fig. 5:**
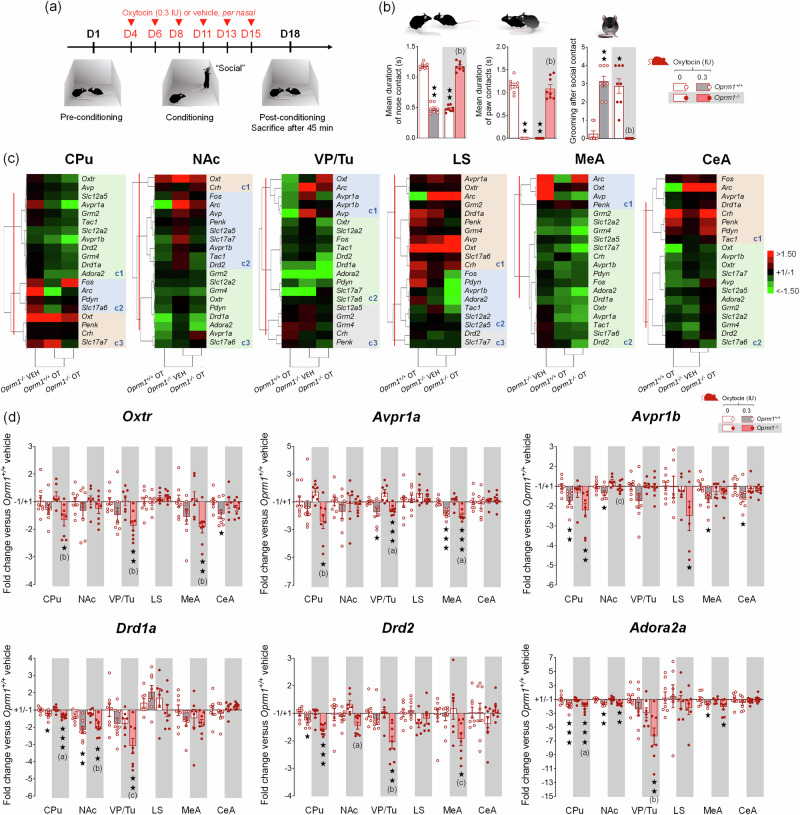
Transcriptional consequences of social OT conditioning in *Oprm1* null mice and their wild-type controls. **a** In this experiment, OT administration was paired with social encounter for all the mice (“social” paradigm; 4 males – 4 females per genotype and treatment). After a pre-conditioning social interaction session, mice received per nasal OT (0.3 IU) or vehicle administration paired with the presentation of an unfamiliar mouse every two/three days over 2 weeks (D4 to D15). The mice performed a post-conditioning social interaction session on D18 and were sacrificed 45 min after the beginning of behavioural assessment for qRT-PCR analysis. **b** As observed in the previous experiment, OT exposure had opposite effects on social interaction in *Oprm1*^*+/+*^ and *Oprm1*^*-/-*^ mice, inducing a severe deficit in the former while rescuing interaction in the latter (mean duration of nose contacts: *H*_*3,32*_ = *23.3, p* < 0.0001*;* mean duration of paw contacts: *H*_*3,32*_ = *26.8, p* < 0.0001*;* grooming after social contact: *H*_*3,32*_ = *25.6, p* < 0.0001) (more parameters in Fig. S5). **c** A hierarchical clustering analysis of qRT-PCR data was performed for each brain region of interest. The most contrasted transcriptional profiles were observed between OT-treated *Oprm1*^*+/+*^ and OT-treated *Oprm1*^*-/-*^ mice in the NAc, VP/Tu, LS, and CeA, but not in the CPu and MeA where OT exposure led to more similar profiles between *Oprm1*^*+/+*^ and *Oprm1*^*-/-*^ mice. The main transcriptional effect of OT was to down-regulate gene expression across brain regions (gene names highlighted in green), as seen in the CPu, NAc, VP/Tu, MeA and CeA, but not in the LS. **d** OT treatment decreased the expression of genes coding for oxytocin and vasopressin receptors (*Oxtr, Avpr1a, Avpr1b*) in the CPu, VP/Tu and MeA, more significantly in *Oprm1*^*-/-*^ than in *Oprm1*^*+/+*^ mice. Similarly, OT exposure led a down-regulation of the expression of the main marker genes of SPNs, the genes coding for the dopamine D1 (*Drd1a*) and D2 (*Drd2*) receptors, and the gene coding for the adenosine 2a (*Adora2*) receptor. Such down-regulation was more pronounced in the VP/Tu of the *Oprm1*^*-/-*^ mice. Gene expression data are expressed as fold change versus *Oprm1*^*+/+*^ - vehicle group (clustering or scatter plots and mean ± SEM). Comparison to *Oprm1*^*+/+*^ - vehicle group (two-tailed t-test): one star *p* < 0.05, two stars *p* < 0.01, three stars *p* < 0.001. Letters: significant difference with vehicle-treated *Oprm1*^*-/-*^ group (2-tailed t-test); (**c**): *p* < 0.05, (**b**): *p* < 0.01, (**a**): *p* < 0.001. qRT-PCR data used for clustering are displayed in Table S2. More individual transcriptional profiles for candidate genes are displayed in Fig. S5.

Equivalent numbers of naive male and female animals were used in each group. Mice were aged 8–10 weeks when behavioural testing started. For acute experiments (Figs. 1 and 2), each behavioural test was performed in an independent cohort of mice. For chronic experiments (Figs. 3–5), behavioural tests were performed successively in the same cohort of mice, and a testing order was chosen to minimize the incidence of anxiety generated by each test on later assays, except for the evaluation of nociceptive thresholds. Indeed, the effects of chronic OT on nociception were assessed in a dedicated cohort of mice (see Fig. 3a). Experiments were conducted and analysed blind to genotype and experimental conditions.

As described previously [57, 58], social behaviour was explored using the direct social interaction and three-chamber tests (classical or modified version, see Supplement 1), stereotyped/perseverative behaviour was assessed by scoring spontaneous motor stereotypies, the number of buried marbles in the marble burying test and by analysing alternation patterns in the Y-maze, and anxiety-like behaviour was evaluated in the novelty-suppressed feeding test. Nociceptive thresholds were evaluated using the tail-immersion test. Finally, we used a a conditioning protocol to assess the influence of social context on OT effects (see Supplement 1).

### Real-time quantitative PCR analysis

Real-time quantitative Polymerase Chain Reaction (qRT-PCR) analysis was performed on brain samples as described previously [46, 58] (see dissection in Fig. S1, supplementary experimental procedures in Supplement 1, and list of probes in Table S1).

### Statistics

Statistical analyses were performed using Statistica 9.0 software (StatSoft, Maisons-Alfort, France). For all comparisons, values of *p* < 0.05 were considered significant. Consistent with previous report [58], when conditions of normality were verified (Shapiro-Wilk test), statistical significance in behavioural experiments was assessed using one to four-way analysis of variance (treatment (T) or dose (D), genotype (G), stimulus (S) and paradigm (P) effects) followed by Tukey’s multiple comparisons test. When these conditions were not fulfilled, we used the non-parametric Kruskal-Wallis analysis of variance followed by 2-tailed t-test to assess differences between groups. Under these conditions, genotype and treatment were collapsed into a single factor, and groups were analysed as independent. When a parameter was measured repeatedly (stimulus effect in the 3-chamber test: toy versus mouse, stranger versus cage-mate), however, non-parametric analysis would not allow post-hoc comparisons; thus, ANOVA was maintained, which may have exaggerated statistical significance. As male and female *Oprm1*^*-/-*^ mice display similar behavioural deficits [45, 49] and preliminary experiments did not reveal differential OT effects between sexes, we pooled male and female data in the present study. However, a principal component analysis (PCA) was performed *a posteriori* on social interaction parameters in OT-treated *Oprm1* null mice to assess whether sex influenced OT effects across experimental conditions. We used nose and paw contact duration, number of following and rearing episodes and number of grooming events after social contact in this analysis. We considered the two first extracted principal components (PC1 and PC2) as accounting for the most relevant variance in the data set and used them for schematic representation. As described previously [46, 58], qRT-PCR data were transformed prior to statistical analysis to obtain a symmetrical distribution centred on 0, using the following formula: if x < 1, y = 1-1/x; if x > 1, y = x-1 (x: qPCR data; y: transformed data). Outliers over twice the standard deviation were excluded from calculations (without iteration), as technical errors. The significance of qRT-PCR data was then assessed using a two-tailed t-test; an adjusted *p* value was calculated using Benjamini-Hochberg correction for multiple testing. Unsupervised clustering analysis was performed on transformed qRT-PCR data using complete linkage with correlation distance (Pearson correlation) for drug, treatment, and brain region (Cluster 3.0 and Treeview software).

## Results

### Acute *per nasal* administration of OT dose-dependently restored social behaviour in *Oprm1* null mice

We first assessed the effects of intranasal OT administration over a range of 3 doses: 0.15, 0.3, and 0.6 IU, on social behaviour in *Oprm1*^*-/-*^ mice and their WT counterparts.

When administered 5 min before behavioural testing, acute intranasal OT modified direct social interaction in *Oprm1* null mice following an inverted U-shaped dose-response curve (Fig. 1a and S2a). Indeed, vehicle-treated *Oprm1*^*-/-*^ mice displayed a deficit in social interaction; this deficit was partially reversed after per nasal OT administration at 0.15 IU, fully at 0.3 IU but failed to be relieved at the dose of 0.6 IU. Negative effects of OT at 0.6 IU dose in *Oprm1*^*+/+*^ mice were detected on the time spent in nose contact. Thus, the dose of 0.3 IU OT was the most efficient to restore social interaction in *Oprm1* null mice, when administered via per nasal route 5 min before testing. When administered 15 min before testing (Fig. 1b and S2b), this dose of OT had partial effects on social interaction in mutant mice. When administered 30 min before testing (Fig. 1c and S2c), acute intranasal 0.3 IU OT had no effect on social interaction. The delay of 5 min after nasal administration was thus used for the next experiments.

We then assessed whether OTR activation is indeed responsible for the effects of intranasal OT on social interaction in *Oprm1*^*-/-*^ and *Oprm1*^*+/+*^ mice. We injected the selective non-peptide OT antagonist LIT183 (pharmacological properties and chemical synthesis in Supplement 1) by intraperitoneal route at 7.5 or 15 mg/kg 25 min before intranasal OT (0.3 IU) administration and 30 min before testing (Fig. 1d and S2d). In *Oprm1*^*-/-*^ mice, OT failed to completely restore social interaction parameters when LIT183 was pre-administered. Thus, increased social interaction after intranasal OT in *Oprm1*^*-/-*^ mice relied on the activation of OT receptors, but likely not exclusively.

To further characterize the effects of acute intranasal OT in *Oprm1* null mice, we assessed the effects of this treatment on social preference and social novelty preference in the 3-chamber test. We used a modified version of the social novelty preference phase however, where the second stranger mouse was replaced by a cage mate. During the social preference phase (Fig. 1e and S2e), intranasal OT fully rescued social preference in *Oprm1*^*-/-*^ mice, with no effects in *Oprm1*^*+/+*^ controls. During the modified social novelty preference phase (Fig. 1f and S2f), WT mice displayed a preference for interacting with a cage mate versus the stranger mouse discovered in the previous phase; intranasal OT completely reversed this preference. In contrast, *Oprm1* null mice failed to discriminate between cage mate and stranger mouse during this phase, and OT increased their interest in the cage mate. Thus, OT restored WT-like social preference and cage mate preference in *Oprm1*^*-/-*^ mice.

### Acute intranasal OT relieved anxiety and induced analgesic effects in *Oprm1* null mice but had little influence on stereotypies and perseveration

We then evaluated the effects of acute per nasal OT administration at 0.15, 0.3, or 0.6 IU on non-social behaviour in *Oprm1*^*-/-*^ mice and their WT counterparts. Regarding spontaneous motor stereotypies (Fig. 2a and S3a), *per nasal* OT, reduced circling episodes in *Oprm1* knockout mice increased this behaviour in *Oprm1*^*+/+*^ mice (0.6 IU). No significant difference between groups was detected in the marble burying test (Fig. S3b). When exploring the Y-maze (Fig. 2c and S3c), 0.15 and 0.6 IU OT-treated *Oprm1*^*-/-*^ mice did not display significant perseverative behaviour, in contrast with vehicle and 0.3 IU OT-treated *Oprm1*^*-/-*^ mice. In the NSF test (Fig. 2d and S3d), intranasal OT normalised the latency to feed in *Oprm1*^*-/-*^ mice.

In the tail immersion test (Fig. 2e), per nasal OT showed analgesic properties at 0.15 and 0.3 IU in *Oprm1*^*-/-*^ mice and at 0.6 IU in *Oprm1*^*+/+*^ and *Oprm1*^*-/-*^ mice at 48 °C. At 50 °C, per nasal OT showed analgesic properties at 0.15 and 0.3 IU in *Oprm1*^*-/-*^ mice. No significant effect of OT was detected in the tail immersion test at 52 °C nor in the hot plate test at 51 °C (Fig. S3e). Taken together, these results indicate that intranasal OT had little effect on stereotyped behaviour in *Oprm1* null mice but normalised their anxiety levels in the novelty-suppressed feeding test and induced analgesia in the tail immersion test.

### Chronic intranasal OT maintained prosocial effects in *Oprm1* knockout mice while produced a severe social deficit in wild-type controls

We then questioned whether the effects of intranasal OT would maintain over repeated daily administration in *Oprm1*^*-/-*^ mice. We administered OT via *per nasal* route at 0.3 IU once daily for 16 days and evaluated behaviour from day 8, starting behavioural tests 5 min after intranasal administration (Fig. 3a).

Focusing first on social behaviour, we observed that repeated per nasal OT administration severely compromised social interaction in *Oprm1*^*+/+*^ controls, leading to deficits of similar amplitude than those observed in vehicle-treated *Oprm1* mutants, but maintained its rescuing effects in *Oprm1*^*-/-*^ mice (Fig. 3b and S4a). Similarly, in the three-chamber test (Fig. 3c and S4b), repeated OT administration dramatically impaired social preference in *Oprm1*^*+/+*^ mice while maintaining its benefits in *Oprm1* mutants. Thus, chronic OT demonstrated prolonged prosocial effects in *Oprm1* null mice, whereas it decreased social behaviour in WT mice.

Regarding motor stereotypies, chronic per nasal OT was ineffective reducing excessive spontaneous circling behaviour in *Oprm1*^*-/-*^ mice (Fig. 3d and S4c). Consistent with this, in the marble burying test, OT failed to normalise marble burying in *Oprm1* mutants (Fig. S4d). In the Y-maze (Fig. 3f and S4e), chronic OT failed to suppress perseverative same arm entries (SAR) in *Oprm1* mutants. In the novelty-suppressed feeding test (Fig. 3g and S4f), chronic intranasal OT did not decrease feeding latency in *Oprm1*^*-/-*^ mice. Together, these results indicate that repeated OT administration was not able to reduce stereotyped and perseverative behaviours or anxiety levels in *Oprm1*^*-/-*^ mice.

We finally explored the analgesic effects of chronic intranasal OT in *Oprm1*^*-/-*^ and *Oprm1*^*+/+*^ mice. In the tail immersion test (Fig. 3h and S4g), *Oprm1*^*-/-*^ mice displayed lowered nociceptive thresholds that were normalised by OT at 48 °C and 50 °C. At 52 °C, chronic OT increased the nociceptive threshold in *Oprm1* mutants. In the hot plate test (Fig. S4h), chronic OT failed to normalize jumping latency in *Oprm1*^*-/-*^ mice. Therefore, repeated OT administration maintained analgesic effects in the tail immersion test for both *Oprm1*^*-/-*^ and *Oprm1*^*+/+*^ mice, but was ineffective in the hot plate test at 51 °C.

### Prosocial effects of repeated intranasal OT in *Oprm1* null mice were greater and lasted longer when associated with social experience

We challenged the social salience hypothesis by evaluating the influence of repeatedly pairing social experience to intranasal OT injection (social paradigm), compared to pairing with presentation of an inert novel object (object paradigm, Fig. 4a). *Oprm1*^*-/-*^ mice and their *Oprm1*^*+/+*^ controls were tested for direct social interaction before receiving 6 administrations of OT (0.3 IU) or vehicle (every 2-3 days from D4 to D15) 5 min before entering an arena with an object or an unfamiliar conspecific. Social interaction was retested post-conditioning (drug free) on D18, D25, and D32. Social preference was evaluated on D20.

During the preconditioning session, *Oprm1*^*-/-*^ mice displayed a severe social deficit compared to *Oprm1*^*+/+*^ mice (Fig. S5a). In contrast, after repeated exposure to intranasal OT (D18, Fig. 4b and S5b), social interaction was severely compromised in *Oprm1*^*+/+*^ mice whilst social deficit was relieved in *Oprm1* knockouts. In mutant mice, however, the prosocial effects of OT were of higher amplitude when mice experienced social encounters immediately after OT administration (social paradigm). One week after the first postconditioning assessment of social interaction (D25, Fig. 4c and S5c), deficient social interaction was still detected in OT-treated *Oprm1*^*+/+*^ mice trained under the “object” and “social” paradigms, while OT treatment maintained prosocial effects in *Oprm1*^*-/-*^ mice only when trained under the “social” paradigm. After another week (D32, Fig. 4d and S5d), a social behaviour deficit was still detected in OT-treated *Oprm1*^*+/+*^ mice; prosocial effects of OT in *Oprm1*^*-/-*^ mice trained under the “social” condition were maintained only for a few parameters. Thus, the effects of intranasal OT on social behaviour in *Oprm1* null mice were greater and longer lasting when this treatment was paired with social experience. The effects of repeated OT exposure were also assessed in the three-chamber test for social preference (D20, Fig. 4e and S5e). To challenge the hypothesis that the conditioning paradigm influences the relieving effects of OT exposure on social preference in the *Oprm1* mouse model of ASD, we focused our analysis on *Oprm1*^*-/-*^ mice and evidenced more significant restoration of social preference parameters under the “social” than the “object” paradigm.

In conclusion, repeated OT exposure better rescued social preference in *Oprm1* knockout mice when this treatment was associated with social experience.

### Transcriptional consequences of social OT conditioning in *Oprm1* null mice and their wild-type controls

To gain insight into the molecular mechanisms at work in the brain of mice that underwent social OT conditioning, we assessed the effects of repeated OT exposure paired with social experience on gene expression 45 min after post-conditioning session (Fig. 5a) in six regions of the reward/social circuit: CPu, NAc, VP/Tu, LS, MeA and CeA in *Oprm1*^*-/-*^ *and Oprm1*^*+/+*^ mice. We focused on genes coding for key players of the oxytocin/vasopressin system, marker genes of SPNs, and neuronal expression and plasticity. We monitored behaviour during post-conditioning session (Fig. 5b) and confirmed previous observation of deleterious effects of intranasal OT exposure in *Oprm1*^*+/+*^ mice contrasting with prosocial effects in *Oprm1*^*-/-*^ mutants.

We performed hierarchical clustering analysis of qRT-PCR data for each brain region to visualize the influence of OT conditioning on gene expression in *Oprm1*^*+/+*^ and *Oprm1*^*-/-*^ mice (Fig. 5c). Transcriptional profiles were more similar between vehicle- and OT-treated *Oprm1*^*-/-*^ mice in the NAc, VP/Tu, LS and CeA, showing predominance of genotype effects; OT treatment led to more similar profiles between OT-treated *Oprm1*^*+/+*^ mice and OT-treated *Oprm1*^*-/-*^ mice in the CPu and MeA. The main transcriptional effect of OT was to down-regulate gene expression across brain regions, as seen in the CPu (cluster1), NAc (cluster3), VP/TU (cluster2), MeA (cluster2), and CeA (cluster2), but not in the LS. Thus, OT treatment globally failed to normalize gene expression in *Oprm1* null mice.

We then focused on candidate genes. We only took into consideration gene expression regulations affecting several brain regions for the same gene or several genes with similar functional profiles. Regarding the oxytocin/vasopressin system, transcriptome analysis revealed a global downregulation of the expression of genes coding for the OT (*Oxtr*) and vasopressin (*Avpr1a*, *Avpr1b*) receptors, in mice of both genotypes, after OT exposure (Fig. 5d). This regulation affected mostly the CPu, VP/Tu and MeA, and was more consistent in *Oprm1* knockouts than in WT controls. In contrast, OT treatment had little influence on the expression of genes coding for oxytocin and vasopressin (Table S2, Fig. S6). Considering SPN markers, OT treatment decreased the expression of genes coding for the dopamine D1 (*Drd1a*) and D2 (*Drd2*) receptors, as well as the adenosine 2a (*Adora2*) receptor, in the CPu, NAc, and VP/Tu for the three genes, and also in the MeA for *Drd2* and *Adora2*. In the VP/Tu, down-regulation was more pronounced in *Oprm1* knockout mice compared to OT-treated WT mice. Thus, transcriptional results indicate that repeated OT exposure suppresses the expression of OT and vasopressin receptors, as well as the expression of the main SPN markers D1, D2, and A2a receptors, with a tendency for more pronounced effects in *Oprm1* null mice compared to wild-type mice.

Finally, we evaluated sex effects on OT modulation of social behaviour in *Oprm1* null mice by performing a PCA on social interaction data across different experimental paradigms (single, chronic, and “object” / “social” conditioning paradigms). This analysis showed no significant effect of sex on social interaction in these mice and illustrated a better normalization under the “social” versus “object” conditioning paradigm (Fig. S7).

## Discussion

The present study extends previous findings in *Oprm1* null mice [50, 51] by showing that the facilitating effects of OT administration on social behaviour in *Oprm1*^*-/-*^ mice tightly depend on the dose tested. Indeed, while administering OT acutely at doses commonly employed in animal and human studies, we observed an inverted U-shaped dose-response curve on social interaction parameters, 0.15 IU being minimal, 0.3 IU producing optimal effects and 0.6 IU being deleterious. Negative effects of a high dose of OT on social behaviour may have resulted from excessive internalisation/uncoupling of OTR [51, 59], activation of vasopressin receptors [60], or recruitment of neural circuits involved in anxiety and fear [61–63]. In comparison, prosocial effects of OT have been detected from the dose of ∼200 µg/kg (0.075 IU) in mice prenatally exposed to sodium valproate or *Cntnap2* null mice [25, 29], suggesting that sensitivity to OT effects may vary between models.

Regarding kinetics, OT effects (0.3 IU) were optimal in *Oprm1*^*-/-*^ mice at a short delay after administration (5 min), consistent with previous findings [40, 50], and vanished rapidly. Accordingly, OT is detected in the brain 5 min after intra-nasal delivery [19, 26, 64]. After intraperitoneal administration, however, rescuing effects of OT on social behaviour in mouse models of ASD have been observed for up to 2 hours after administration [25, 29], likely due to persistent high blood levels of OT reached using this route [19]. In the present study, we focused on intranasal route as poorly invasive; it reveals, however, some limitations regarding the duration of OT effects. As concerns frequency of administration, increased social interaction was maintained under repeated OT treatment in *Oprm1*^*-/-*^ mice, as demonstrated in other ASD models [27, 30]. In contrast, such treatment severely and long-lastingly impaired social behaviour in wild-type mice, consistent with a previous report [40]. Together, our results highlight the critical importance of the choice of dose and timing of OT administration for therapeutic use in the context of ASD. Moreover, a well-established ASD diagnostic appears a critical prerequisite to OT treatment, considering the social deficit induced by chronic OT in neurotypical subjects.

Improved social interaction following acute OT administration in *Oprm1*^*-/-*^ mice involved OTR, as this effect was reduced in the presence of the selective non-peptide OTR antagonist LIT183. However, a contribution of V_1A_ and V_1B_ vasopressin receptors is likely to account for the lack of complete reversal observed at a high dose of LIT183. Indeed, OT and vasopressin can bind to each other’s receptors to improve sociability [17, 65, 66].

We further explored OT effects in *Oprm1* null mice by assessing social preference. A single intranasal injection of OT restored preference for interacting with a congener over a mouse-shaped inert toy, likely by facilitating a social approach [9, 67]. Interestingly, in *Oprm1* null mice, OT also restored preference for a familiar conspecific in our modified version of the social novelty phase, as observed in vehicle-treated *Oprm1*^+/+^ mice. Here, OT administration likely facilitated social memory and discrimination in *Oprm1* mutants [11, 32]. Conversely, OT administration reoriented social preference towards the most novel congener in *Oprm1*^+/+^ mice. This effect of OT in wild-type mice may be attributable to an attenuation of social fear or vigilance together with an increase in social approach, disinhibiting exploration of a novel conspecific [67–70]. Therefore, OT demonstrated prosocial effects in *Oprm1* null mice, consistent with previous results in other murine models of ASD. Of note, the brain oxytocin system was found altered in all these models [26, 29, 71] as in *Oprm1* null mice, raising the hypothesis that oxytocin deficits are a prerequisite to successful OT treatment in ASD.

In our study, a single OT injection had little effect on the non-social dimension of ASD-like deficits in *Oprm1*^*-/-*^ mice, namely stereotyped behaviours and perseveration, as previously reported for the *Cntnap2*^*-/-*^ ASD mouse model [29]. However, improvements in stereotypies or cognitive flexibility have been observed in other models when OT administration was repeated [17, 72] or performed early in life [26, 71]. Similarly, in subjects with ASD, OT administration was either reported to decrease restricted/repetitive behaviour [73, 74] or to be inefficient in this dimension [33]. Such inconsistencies may reflect the recruitment of OT-sensitive brain substrates with antagonistic effects on repetitive behaviours, depending on the dose and frequency of administration [75, 76]. Consistent with anxiolytic properties [77], acute OT normalised anxiety levels in *Oprm1* mutants. This effect, however, was lost under repeated administration, maybe due to the anxiogenic effects of OT developed under chronic administration [62]. In contrast, analgesic effects of OT on spinal nociception in *Oprm1*^*-/-*^ mice were maintained upon chronic administration, suggesting the involvement of differential neuronal substrates [78, 79]. Thus, OT treatment appears more efficient on the core, social, dimension of ASD symptoms than stereotypies or secondary symptoms.

The main finding of our study was that the prosocial effects of OT in *Oprm1*^*-/-*^ mice were greater and longer lasting when this neuropeptide was administered in a social context. These results are in line with a growing body of literature pointing towards social environment as a key determinant for the prosocial effects of OT [42, 43]. OT therefore appears to behave as a coincidence detector for social experience and reward processes, allowing the reinforcement of social interactions. The neuronal substrate of such action would involve striatal regions, notably D_1_R and D_2_R-SPNs in the NAc, and interaction with multiple neuromodulators [9, 80]. In mice exposed to OT concomitantly with social experience, we found *Drd1a* and *Drd2* transcripts downregulated in the CPu, as well as in the NAc of *Oprm1*^*+/+*^ and *Oprm1*^*-/-*^ mice for the former, and in the VP/Tu of *Opmr1* null mice for the latter, pointing to modulation of dopaminergic transmission in these regions irrespective of genotype [81]. Interestingly, the expression of *Adora2a*, coding for adenosine A_2a_ receptors, was also consistently down-regulated in the striatum of *Oprm1* wild-type and mutant mice, and in the VP/Tu for *Opmr1* null mice. Such downregulation affected specifically D_2_R-expressing SPNs, of which *Adora2a* is a selective gene marker [82]. Not only A_2a_ receptors activate D_2_-SPNs but they also inhibit D_2_R signalling through heterodimerization [83]. Thus, decreased *Adora2a* expression may have facilitated a reduction in NAc D_2_R-SPN activity in *Oprm1* deficient mice, then facilitating social approach [58]. The contribution of D_1_R and D_2_R-SPNs to the social context-driven effects of OT deserves further exploration.

Interestingly, the expression of *Oxtr*, *Avpr1a*, and *Avpr1b*, coding for OT and V_1a_ and V_1b_ vasopressin receptors, was also downregulated, a likely adaptive consequence of repeated exogenous OT administration. Decreased *Oxtr* expression in wild-type mice was previously reported after chronic intranasal OT (twice a day for 7 days) and proposed to contribute to the adverse effects of this treatment on social behaviour [40]. In our study, six OT injections triggered severe social deficit in *Oprm1*^*+/+*^ mice but no marked regulation of *Oxtr* expression; in contrast, decreased *Oxtr* transcription was detected in *Oprm1*^*-/-*^ mice, in which the prosocial effects of chronic OT were preserved. Thus, *Oxtr* regulation of expression may not have played a major role in mediating the social effects of OT in *Oprm1*^*+/+*^ and *Oprm1*^*-/-*^ mice. Experiments assessing receptor protein levels would be required, however, to confirm this hypothesis [84].

There are several limitations to our investigation of oxytocin/vasopressin gene expression. In the present study, we failed to detect decreased *Oxt* levels in the NAc in *Oprm1*^*-/-*^ mice, in contrast with previous reports [45, 49]. However, under the social paradigm used for qRT-PCR experiments, mutant mice had repeatedly interacted with a wild-type congener, which we showed to increase NAc *Oxt* expression [49]. As such, it is likely that the downregulation of *Oxt* transcripts in the NAc of *Oprm1* mutant mice was counteracted by the effect of repeated social interaction. Futhermore, we did not evaluate *Oxt* transcript levels directly in source regions, namely the PVN and SON. Future studies using a more sensitive method than qRT-PCR, such as single cell transcriptomics, would be useful to further assess the impact of OT treatment on brain *Oxt* expression. Finally, although modified *Oxtr*, *Avpr1a* and *Avpr1b* levels argue for central effects of OT, we cannot exclude that a peripheral action contributed to behavioural changes.

In conclusion, our study provides several insights to better understand discrepancies in the results of recent clinical trials for OT in ASD [35]. First, this work highlights the crucial role of social context for OT effects. Consistent with our findings, when intranasal OT administration in children with ASD was immediately followed by positive social interaction, significant behavioural improvements were measured after a 6-week treatment, with the use of the gold standard ADOS-2 evaluation [85]. Together, these results strongly argue for combining OT administration with behavioural intervention [86]. Then, one may consider the use of a standard dose of OT in ASD as questionable and propose the re-evaluation of the therapeutic dose in future clinical studies. Different aetiologies, alterations in the OT system and/or reward circuit [87] as well as *OXTR* SNP variants may require adapting OT dose individually.

## Supplementary information

Supplemental material

## Data Availability

Data will be made available on request.
