# Supplementary material for: Acute, chronic and conditioned effects of intranasal oxytocin in the mu-opioid receptor knockout mouse model of autism: Social context matters
Source: Neuropsychopharmacology. 2024 Jul 17;49(12):1934–46. doi: 10.1038/s41386-024-01915-1 (PMC11473707; doi:10.1038/s41386-024-01915-1)
Supplement: Supplementary file 1 — Supplemental material [file 41386_2024_1915_MOESM1_ESM.pdf]

Acute, chronic and conditioned effects of intranasal oxytocin in the mu opioid receptor knockout mouse model of autism: social context matters

Fani Pantouli, Camille Pujol, Cécile Derieux, Mathieu Fonteneau, Lucie P Pellissier, Claire Marsol, Julie Karpenko, Dominique Bonnet, Marcel Hibert, Alexis Bailey, Julie Le Merrer*, Jerome AJ Becker*

Supplementary methods

Breeding procedures and housing conditions

Oprm1$^{+/+}$ and *Oprm1*$^{-/-}$ pups (F3) were bred from homozygous parents (F2), as we previously showed that parental care has no influence on behavioural phenotype in these animals (cross-fostering experiments [1]). Homozygous parents were bred from heterozygous animals (F1), to prevent genetic derivation. Mice in the same cage were of the same genotype: this breeding scheme likely exacerbates behavioural deficits in mutant animals by maintaining them together during early post-natal development [2]. Cardboard igloos (Dietex®, Argenteuil, France) and laying were provided in each cage as enrichment. Routine veterinary care and animals' maintenance was provided by dedicated and trained personnel. At the end of experiments, mice were killed either by exposure to rising concentrations of CO_2 over 5 min or cervical dislocation when brain samples were collected (qRT-PCR experiments).

Behavioural testing

Social abilities

Direct social interaction test. The experimental protocol was adapted from [3,4]. On testing day, a pair of unfamiliar mice (not cage mates, age-, sex-, genotype- and treatment-matched) was introduced in one of 4 square arenas (50 x 50 cm, separated by 35 cm-high opaque grey Plexiglas walls) over a white infrared floor (View Point, Lyon, France) for 10 min (15 lx). Each arena received a black plastic floor (transparent to infrared) to minimize anxiety levels. The total amount of time spent in nose contact (nose-to-nose, nose-to-flank and nose-to-anogenital region), the number of these

contacts, the time spent in paw contact and the number of these contacts, grooming episodes (allogrooming), notably ones occurring immediately (<5 s) after a social contact (considered a sign of social discomfort, robustly increased in mouse models of ASD [5,6]), as well as the number of following episodes were scored a posteriori on video recordings (infrared light-sensitive video camera) using an ethological keyboard (Labwatcher®, View Point, Lyon, France) by trained experimenters, and individually for each animal. The mean duration of nose and paw contacts was calculated as the number of events divided by the total time spent in these events. Thus, these measures were normalized regarding the number of events and were preferred for displaying in main figures. Of note, paw contacts were the most difficult social interaction parameter to restore pharmacologically in previous studies, and disappeared first when treatment effects vanished [5,6].

Three-chamber social preference test. The experimental protocol was adapted from [3,7]. The test apparatus consisted of a grey external Plexiglas box with transparent partitions dividing the box into three equal chambers (40 x 20 x 22.5 cm). Two sliding doors (8 x 5 cm) allowed transitions between chambers. Cylindrical wire cages (18 x 9 cm, 0.5 cm diameter-rods spaced 1 cm apart) were used to contain the mouse interactors and object (soft-toy mouse) placed in the two outward chambers of the 3-chamber social preference test. The test was performed under low-light conditions (15 lx) to reduce anxiety. Stimulus wild-type mice were habituated to wire cages for 2 days before the test (20 min/day). On testing day, the experimental mouse was introduced to the middle chamber and allowed to explore the whole apparatus for a 10-min habituation phase (wire cages empty). For the social preference phase, the experimental mouse was confined back in the middle-chamber while the experimenter introduced an unfamiliar wild-type age and sex-matched mouse (8-14-week-old, grouped housed) into a wire cage in one of the side-chambers and a soft toy mouse (8 x 10 cm) in the second wire cage. Then the experimental mouse was allowed to explore the apparatus for 10 min. For the modified novelty preference phase, the experimental mouse was returned to the middle chamber and the soft toy was replaced by a cage mate, to offer the choice to the experimental mouse of interacting whether with a very familiar mouse, the cage mate, or with the congener met during the first phase of the test. The sliding doors were reopened allowing an additional 10-min exploration. The time spent in each chamber and in nose contact with each wire cage, the number of these contacts and the number of entries in each chamber were scored a posteriori on

video recordings using an ethological keyboard (Labwatcher®, View Point, Lyon, France) by trained experimenters. The mean duration of nose contacts (nose to nose, nose to flank, nose to anogenital region) was calculated from these data [3,4]. Preference ratio was calculated as follows: Time in nose contact with the mouse / (Time in nose contact with the mouse + Time in nose contact with the object) x 100. The relative position of stimulus mice was counterbalanced between groups.

Stereotyped behaviours

***Motor stereotypies*.** The experimental protocol was adapted from [8]. To detect spontaneous motor stereotypies in mutant versus wild-type animals, mice were individually placed in clear standard home cages (21×11×17 cm) filled with 3-cm deep fresh sawdust for 10 min. No water was available. Light intensity was set at 30 lux. Trained experimenters scored numbers of spontaneous head shakes, rearing, burying, self-grooming and circling episodes and the total amount of time spent burying by direct observation.

***Marble-burying*.** Marble burying was used as a measure of stereotyped/perseverative behaviour [7,9]. Mice were introduced individually in transparent cages (21×11×17 cm) containing 20 glass marbles (diameter: 1.5 cm) evenly spaced on 4-cm deep fresh sawdust. To prevent escapes, each cage was covered with a filtering lid. Light intensity in the room was set at 40 lx. The animals were removed from the cages after 15 min, and the number of marbles buried more than half in sawdust was recorded.

***Y-maze exploration*.** Spontaneous alternation behaviour was used to assess perseverative behaviour [10,11]. Each Y-maze (Imetronic, Pessac, France) consisted of three connected Plexiglas arms (15x15x17 cm) covered with distinct wall patterns (15 lx). Floors were covered with lightly sprayed fresh sawdust to limit anxiety. Each mouse was placed at the centre of a maze and allowed to freely explore this environment for 5 min. The pattern of entries into each arm was quoted on video-recordings. Spontaneous alternations (SPA), i.e. successive entries into each arm forming overlapping triplet sets, alternate arm returns (AAR) and same arm returns (SAR) were scored, and the percentage of SPA, AAR and SAR was calculated as following: total SPA or AAR or SAR / (total arm entries -2) * 100.

Anxiety-like behaviour

***Novelty-suppressed feeding*.** The protocol was adapted from [12]. Novelty-suppressed feeding (NSF) was measured in 16-hr food-deprived mice, isolated in a standard housing cage for 30 min before individual testing. This test was performed in

the same arenas as the ones used for direct social interaction. Three pellets of ordinary lab chow were placed on a white tissue in the centre of each arena, lit at 60 lx. Each mouse was placed in a corner of an arena and allowed to explore for a maximum of 15 min. Latency to feed was measured as the time necessary to bite a food pellet. Immediately after an eating event, the mouse was transferred back to home cage (free from cage-mates) and allowed to feed on lab chow for 5 min. Food consumption in the home cage was measured.

Nociceptive thresholds

***Tail-immersion test*.** This test was performed as previously described [13,14]. Nociceptive thresholds were assessed by immersing the tail of the mice (5 cm from the tip) successively into water baths at 48°C, 50°C and 52°C. Mice were gently maintained in a pocket during this experiment. The latency to withdraw the tail was measured at each temperature, with a cut-off of 10 s.

Oxytocin conditioning protocol

Animals were randomly distributed across four experimental conditions before behavioural assays had started: saline-object paradigm; OT 0.3 IU-object paradigm; saline-social interaction; OT 0.3 IU-social paradigm (see timeline in Figure 4).

Pre-conditioning: Mice were evaluated for their basal social behaviour at day 1 (D1) of the protocol, using the direct social interaction test (10 min, protocol as described above).

Conditioning: Animals underwent 6 conditioning sessions at D4, 6, 8, 11, 13 and 15. During each conditioning session either OT (intranasal route, 0.3 IU) or saline were administered 5 min before a 10 min interacting session with either a novel mouse (age and sex-matched; “social” paradigm) or a novel object (“object” paradigm; dice, marble, Lego® brick, miniature plastic gem/rock/tree, wooden clip) each time, accordingly to the randomization group.

Post-conditioning: Drug-free mice undergone a direct social interaction test (10 min) at D18 and a three-chamber test for social preference (two phases of 10 min) at D20. To assess the maintenance of OT conditioning over time, two additional sessions of social interaction (10 min) were performed at D25 and 32.

A cohort of mice (social conditioning paradigm only) was dedicated to qRT-PCR analysis and sacrificed 45 min after the direct social interaction test on D18.

Supplementary methods: Real-time quantitative PCR analysis

Brains were removed and placed into a brain matrix (ASI Instruments, Warren, MI, USA). Caudate putamen (CPu), nucleus accumbens (NAc), ventral pallidum/olfactory tubercle (VP/Tu), lateral septum (LS) and central amygdala (CeA) were punched out while medial amygdala (MeA) was dissected from 1mm-thick slices (see Figure S1). Tissues were immediately frozen on dry ice and kept at -80°C until use. For each structure of interest, genotype and condition, samples were processed individually (n=8). RNA was extracted and purified using the Direct-Zol RNA MiniPrep kit (Zymo research, Irvine, USA). cDNA was synthesized using the ProtoScript II Reverse Transcriptase kit (New England BioLabs, Évry-Courcouronnes, France). qRT-PCR was performed in quadruplets on a CFX384 Touch Real-Time PCR Detection System (Biorad, Marnes-la-Coquette, France) using iQ-SYBR Green supermix (Bio-Rad) kit with 0.25 µl cDNA in a 12 µl final volume in Hard-Shell Thin-Wall 384-Well Skirted PCR Plates (Bio-rad). Gene-specific primers were designed using Primer3 software to obtain a 100- to 150-bp product and purchased from Sigma-Aldrich (Saint Quentin, France); sequences are displayed in Table S1. Relative expression ratios were normalised to the expression level of actin and the $2^{-\Delta\Delta Ct}$ method was applied to evaluate differential expression level. We focused primarily on genes coding for key players of the oxytocin/vasopressin system, peptides (*Oxt, Avp*) and receptors (*Oxtr, Avr1a, Avr1b*). We also measured the expression of marker genes of striatal projection neurons (SPNs) (*Drd1a, Pdyn, Tac1, Crh, Grm2, Drd2, Penk, Adora2, Grm4, Slc12a2, Slc12a5, Slc17a6, Slc17a7*) as well as markers of neuronal expression and plasticity (*Fos*, *Arc*), whose expression was found regulated in *Oprm1* knockout mice [1,2,5].

Chemistry: LIT183, OTR antagonist

Structure:

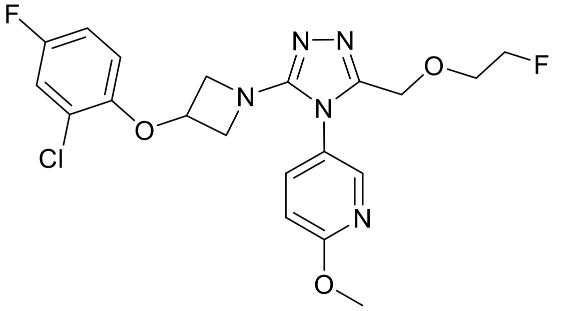

Molecular mass: 451.85 g/mol
HR-MS (ES) [M]: calculated 451.1223, observed 451.1235
HPLC: Tr = 4.33 min, HPLC purity: 100% at 220 nm. Gradient from 5 to 100% of CH_3CN (0.1% TFA) in H_2O (0.1 % TFA), 7.4 min at 1.6 mL/min, Ascentis Express C18 column (2.7 µm, 4.6 mm × 75 mm).

logD (calc) = 2.58

Thermodynamic solubility: 87.9 µg/mL in physiological serum (NaCl 0 .9%) at room temperature.

Affinity (TR-FRET, on HEK293 cells, n=3)[1]:

- OTR: Ki = 1.6 nM
- V1aR: Ki = 1364 nM
- V2R: Ki = 1469 nM

Functional efficacy:

	Functional evaluation				Notes
	**OXTR**	**V1aR**	**V1bR**	**V2R**	
**PF3274167** K_i	9.5	1120	>10000	>10000	Brown et al. 2010[2]
**PF3274167** IC_{50}	8.9 ± 8.7	392 ± 41	nd	nd	N=3, Calcium (Fluo4)[3]
**LIT183** IC_{50}	3 ± 2	360 ± 340	nd	nd	N=2, Calcium (Indo-1)[3]
**LIT183** IC_{50}	10 ± 6.6	390 ± 56	nd	nd	N=3, Calcium (Fluo4)[3]

nd: not detectable

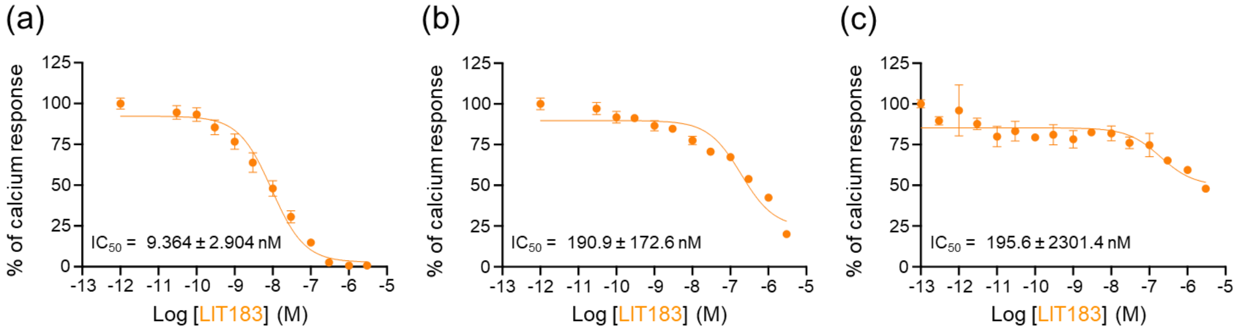

Inhibition dose-response curves of LIT183 (a) on OTR, in presence of oxytocin (10 nM), (b) on V1AR, in presence of vasopressin (1 nM) and (c) on V2R in presence of vasopressin (100 nM). Antagonist properties of LIT183 were determined by measuring intracellular calcium flux in HEK293 cells stably expressing the human oxytocin OT, vasopressin V_{1a} or V_2 receptors [15]. Fluorescence of Fluo4 was recorded at 520 nm (excitation wavelength at 494 nm) for 3 minutes following addition of ligand using FlexStation®III reader (Molecular Devices). Based

on these results, a dose of 15 mg/kg was chosen in vivo to ensure sufficient blockade of OT activity. An additional dose of 7.5 mg/kg allowed a better framing of LIT183 efficacy in mice (similar dose as for L-369,899, IC_{50}=8.9 nM [16]).

Synthesis:

Synthetic scheme:

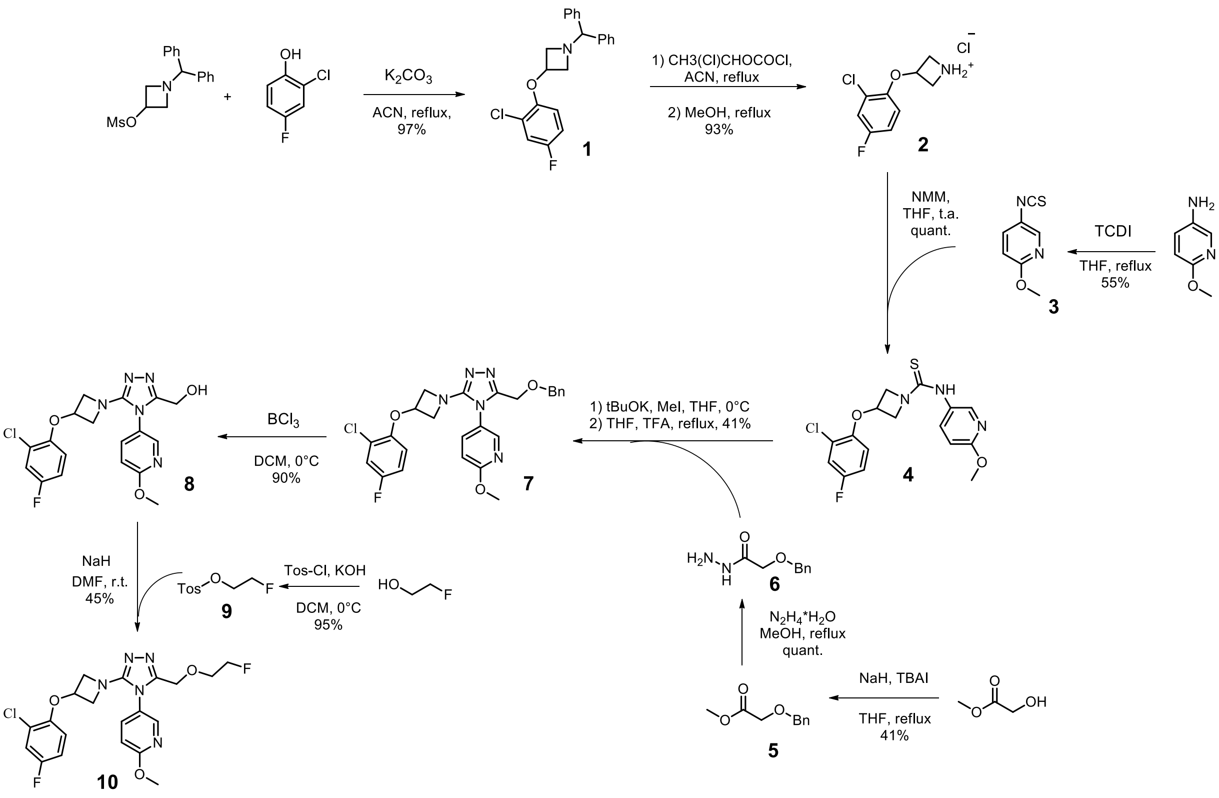

Compound 1:[4] To a solution of 1-(diphenylmethyl)azetidin-3-yl methanesulfonate (3.6 mmol, 1.14 g) and 2-chloro-4-fluorophenol (3.1 mmol, 460 mg) in dry ACN (20 mL) K_2CO_3 (7.5 mmol, 1.08 g) is added. The reaction mixture is stirred under reflux for 4 h. Water (150 mL) is added and the resulting mixture is extracted with CH_2Cl_2 (150 mL). The organic layer is washed with water (2x150 mL), brine (150 mL) and the volatiles are removed under vacuum. The residue is purified by reverse phase flash chromatography eluted with ACN/H_2O (5% to 100% in 30 min) to give **1** as yellow oil (1.1g, 97%).

Compound 2:[5] To a stirred solution of **1** (0.87 mmol, 320 mg) in dry CH_3CN (5 mL) at 0°C 1-chloroethyl chloroformate (1.74 mmol, 188 µL) is added dropwise. The reaction mixture is stirred at 80°C for 1h monitored by HPLC then cooled down to r.t. and concentrated under reduced pressure. The residue is dissolved in dry MeOH (5 mL) and stirred at 65°C for 3h monitored by HPLC. Water (50 mL) is added and the solution is washed with heptane (3x50 mL), then the volatiles are removed under vacuum to give **2** as white solid (193 mg, 93%).

Compound 3:[6] A solution of 5-amino-2-methoxypyridine (0.5 mmol, 40.2 µL) in dry THF (3 mL) is added dropwise over the period of 40 min to a stirred solution of 1,1'-thiocarbonyldiimidazole (0.75 mmol, 148.5 mg) in dry THF (2 mL) cooled to ice-water temperature. The reaction mixture is allowed to warm to room temperature then stirred for 30 min. THF is evaporated, the residue is dissolved in CH_2Cl_2, washed with $NaHCO_3$ (sat), brine then concentrated under vacuum and filtered through a silica gel pad, washed with CH_2Cl_2. The solvent is removed under vacuum to give **3** as white solid (46 mg, 55%).

Compound 4: To a suspension of **2** (3 mmol, 707 mg) in dry THF (15 mL) at 0°C, NMM (3.6 mmol, 392 µL) is added. A solution of **3** (3 mmol, 494 mg) in dry THF (15 mL) is added dropwise. The reaction mixture is heated to r.t. and stirred for 1h. The resulted mixture is concentrated under vacuum. To the residue DCM (150 mL) is added and the organic fraction is washed with water (3x150 mL), brine (150 mL), dried over Na_2SO_4. The volatiles are removed under vacuum to give **4** as white powder (1.1 g, quantitative).

Compound 53:[4] To a solution of methyl glycolate (5 mmol, 396 µL) in dry THF (25 mL) under Ar at 0°C, NaH (5.25 mmol, 60% dispersion in oil, 210 mg) is added. The resulted mixture is stirred at 0°C for 30 min, then NBu_4I (0.5 mmol, 185 mg) is added followed by BnBr (5 mmol, 610 µL). The reaction mixture is heated at 60°C for 24h. Water (100 mL) is added and the mixture is extracted with CH_2Cl_2 (2x50 mL). The combined organic fractions are washed with water (2x100 mL) and brine (100 mL). The volatiles are removed under vacuum and the residue is purified consequently by flash chromatography eluted with EA/heptane (5% for 5 min, then from 5% to 40% in 15 min) and by reverse phase flash chromatography eluted with ACN/H_2O (from 20% to 100% over 30 min) to give **5** as yellow oil (41%).

Compound 6: To a solution of **5** (2.5 mmol, 450 mg) in dry MeOH (20 mL) hydrazine monohydrate (3.7 mmol, 185 µL) is added. The reaction mixture is refluxed for 6h, then the volatiles are removed under vacuum, co-evaporated 3 times with new portions of MeOH, the residue is dried under high vacuum to give the desired compound as colourless oil (448 mg, quantitative).

Compound 7: To a stirred solution of **4** (1.2 mmol, 441 mg) in dry THF (17 mL) cooled to ice-water temperature tBuOK (1.44 mmol, 170 mg) is added. The resulting mixture is stirred for 5 min then MeI (1.44 mmol, 90 µL) is added. The reaction mixture is stirred at ice-water temperature for 15 min. Water (150 mL) is added and the resulted mixture is extracted with EA (2x70 mL). The combined organic fractions are washed with water (3x150 mL), brine (150 mL), dried over Na_2SO_4 and the volatiles are removed under vacuum. The residue is dissolved in dry THF (17 mL). To the obtained solution **6** (1.43 mmol, 257 mg) is added followed by TFA (0.59 mmol, 44 µL). The reaction mixture is stirred under reflux for 6h followed by HPLC, then

the volatiles are removed under vacuum. To the residue water (100 mL) is added and the mixture is extracted with DCM (2x50 mL). The organic layer is washed with water (2x100 mL), brine (100 mL) and dried over Na_2SO_4. The volatiles are removed under vacuum and the residue is purified by reverse phase flash chromatography eluted with ACN (with 0.1% TFA) / H_2O (with 0.1% TFA) (5-100% over 30 min) to give **7** (300 mg, 41%) as yellow oil.

Compound 8: To a solution of **7** (0.11 mmol, 68 mg) in dry DCM (5 mL) cooled to ice-water temperature a solution of BCl_3 in DCM (1 M, 0.56 mmol, 56 µL) is added. The resulting mixture is stirred for 30 min. Water (50 mL) and DCM (50 mL) are added. A solution of 10N NaOH was added dropwise until the pH of the aqueous phase was 13. The organic fraction is separated, washed with 1N solution of $NaHCO_3$ (50 mL), brine (50 mL), dried over Na_2SO_4 and the volatiles are removed under vacuum. The residue is purified by reverse phase flash chromatography eluted with ACN/H_2O (5% to 100 % in 30 min) to give after lyophilization compound **8** as yellow solid (230 mg, 90%).

Compound 9: To a solution of 2-fluoroethan-1-ol (0.5 mmol, 29 µL) in dry DCM (5 mL) cooled to ice-water temperature KOH (1.5 mmol, 84 mg) and Tos-Cl (0.55 mmol, 107 mg) are added. The reaction mixture is stirred at 0°C for 2h then at r.t. overnight. Water (100 mL) and DCM (50 mL) are added. The organic fraction is separated, washed with water (2x50 mL), brine (50 mL), dried over Na_2SO_4 and the volatiles are removed under vacuum. The residue is purified by flash chromatography eluted with EA/heptane (5% - 100% over 30 min) to give **9** as colourless oil (105 mg, 96%).

Compound 10: To a solution of **8** (0.038 mmol, 20 mg) in dry DMF (1 mL) under Ar, KOH (0.19 mmol, 11 mg) is added. The resulted mixture is stirred at 0°C for 10 min, then **9** (0.058 mmol, 12.6 mg) is added. The reaction mixture is stirred at ice-water temperature for 3h, then at r.t. overnight. Water (100 mL) and DCM (50 mL) are added. The organic fraction is separated, washed with water (2x50 mL), brine (50 mL), dried over Na_2SO_4 and the volatiles are removed under vacuum. The residue is purified by reverse phase flash chromatography eluted with ACN/H_2O (5% to 100 % in 30 min) to give after the lyophilization **10** as white solid (15 mg, 69%).

References (chemistry)

1- Karpenko IA, Margathe JF, Rodriguez T, Pflimlin E, Dupuis E, Hibert M, Durroux T, Bonnet D. Selective nonpeptidic fluorescent ligands for oxytocin receptor: design, synthesis, and application to time-resolved FRET binding assay. J Med Chem. **2015**, 58, 2547-2552.

2- A. Brown, D. Ellis, C. Smith, Substituted triazole derivatives as oxytocin antagonists, **2005**, U.S. Patent WO2005028452.

3- Frantz MC, Pellissier LP, Pflimlin E, Loison S, Gandía J, Marsol C, Durroux T, Mouillac B, Becker JAJ, Le Merrer J, Valencia C, Villa P, Bonnet D, Hibert M. LIT-001, the First Nonpeptide Oxytocin Receptor Agonist that Improves Social Interaction in a Mouse Model of Autism. J Med Chem. **2018** 61, 8670-8692.

4- M. J. Fray, P. Allen, P. R. Bradley, C. E. Challenger, M. Closier, T. J. Evans, M. L. Lewis, J. P. Mathias, C. L. Nichols, Y. M. Po-Ba, et al., Tetrahedron **2006**, 62, 6869–6875.

5- A. Brown, A. Calabrese, D. Ellis, 1, 2, 4-Triazole Derivatives and Their Use as Oxytocin Antagonists, **2006**, U.S. Patent US 2011/0092529 A1.

6- A. A. Michrowska-Pianowska, M. Kordes, J. Hutzler, T. W. Newton, R. R. Evans, K. Kreuz, K. Grossmann, T. Seitz, A. van der Kloet, M. Witschel, et al., Herbicidal isoxazolo pyridines, **2013**, U.S. patent WO2013104561.

Supplementary figures

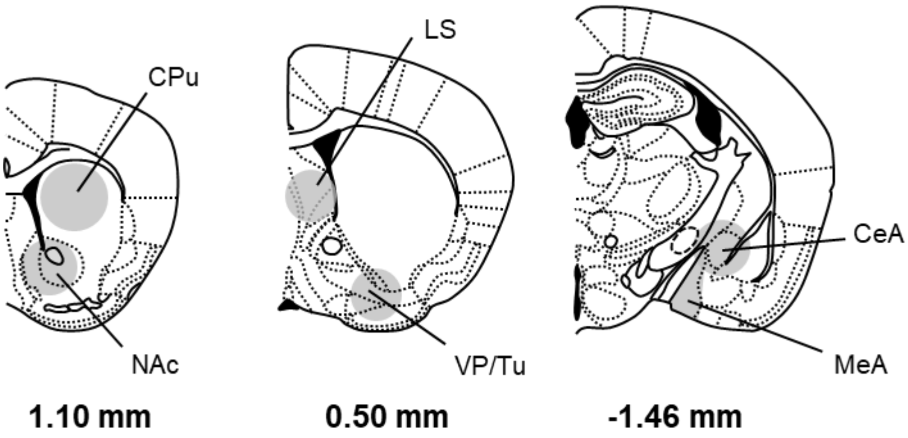

Figure S1. Schematic representations depict brain regions dissected for gene expression study. CPu, NAc, LS, VP/Tu, MeA and CeA, were punched on 1-mm thick brain slices (CPu: one punch/side, ⌀ 2 mm; NAc, BNST, VP, CeA, and VTA/SNc: one punch/side, ⌀ 1.25 mm). Coordinates refer to bregma. CPu: Caudate Putamen; CeA: Central Amygdala; LS: lateral septum; NAc: Nucleus Accumbens; VP/Tu: ventral pallidum/olfactory tubercle.

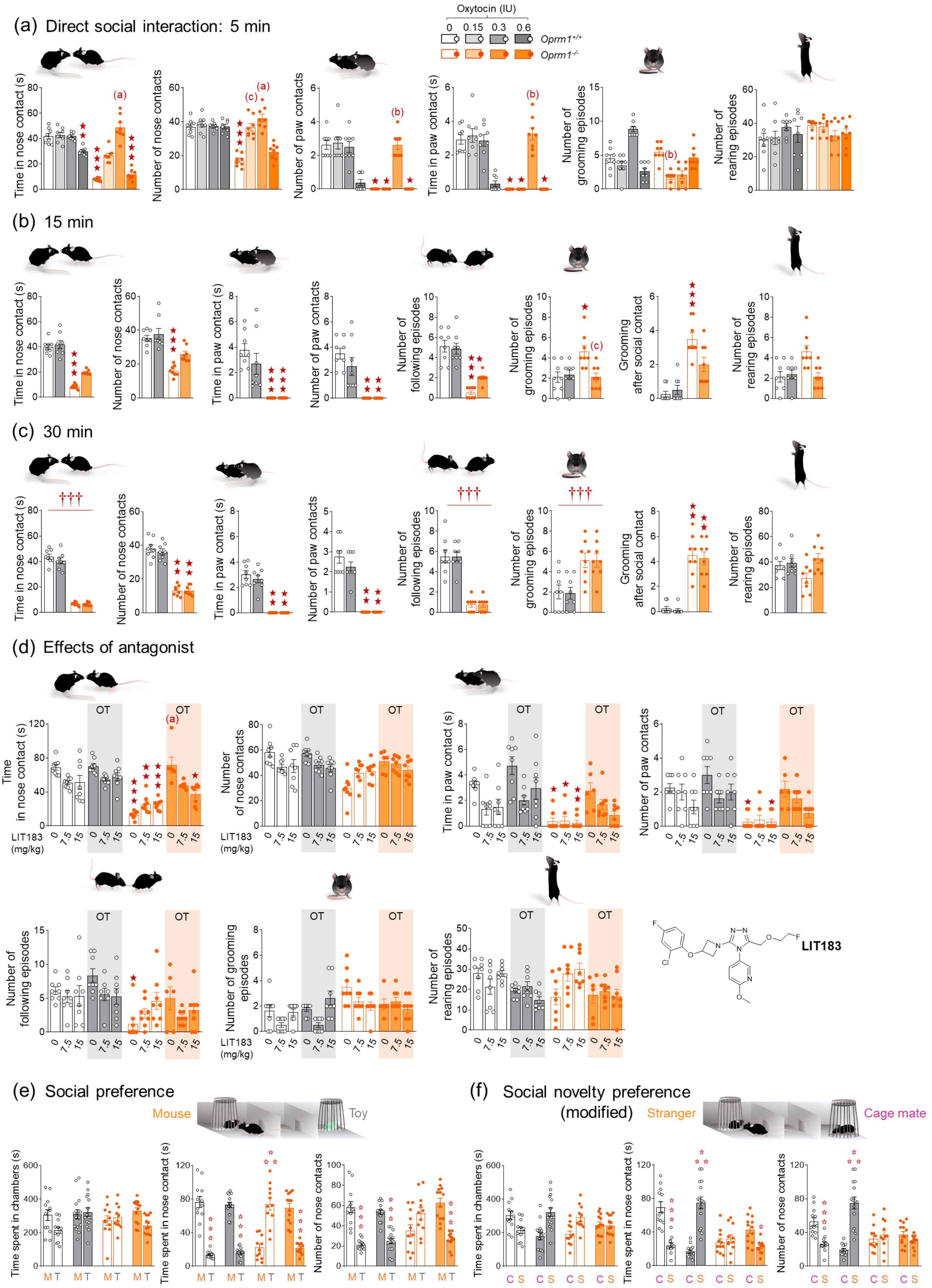

(a) Direct social interaction: 5 min
Oxytocin (IU)
0 0.15 0.3 0.6
Oprm1+/+
Oprm1-/-
Time in nose contact (s)
Number of nose contacts
Number of paw contacts
Time in paw contact (s)
Number of grooming episodes
Number of rearing episodes
(b) 15 min
Number of following episodes
Grooming after social contact
(c) 30 min
(d) Effects of antagonist
OT
LIT183 (mg/kg)
0 7.5 15
LIT183
(e) Social preference
Mouse
Toy
Time spent in chambers (s)
Time spent in nose contact (s)
Number of nose contacts
M T
(f) Social novelty preference (modified)
Stranger
Cage mate
C S

Figure S2. Acute per nasal administration of OT dose-dependently restored social behavior in *Oprm1* null mice. (a) *Oprm1*$^{+/+}$ and *Oprm1*$^{-/-}$ mice received OT or vehicle (4 males – 4 females per genotype and treatment) via per nasal route 5 min before the direct social interaction test at the dose of 0, 0.15, 0.3 or 0.6 IU. Vehicle-treated *Oprm1*$^{-/-}$ mice displayed a severe deficit in social interaction parameters ; OT at 0.15 and 0.3 IU fully restored the time spent in (*Genotype [G] x Dose [D] interaction:* $F_{1,56}=39.80$, $p<0.0001$) and number of nose contacts (*Genotype [G] x Dose [D] interaction:* $F_{1,56}=22.19$, $p<0.0001$) while only the dose of 0.3 IU restored the time spent in ($H_{7,64}=52.71$, $p<0.0001$) and number of paw contacts ($H_{7,64}=53.72$, $p<0.0001$) and time spent in ($H_{7,64}=52.71$, $p<0.0001$) paw contact in mutant mice. Moreover, OT at 0.3 IU decreased the number of grooming episodes in *Oprm1*$^{-/-}$ mice ($H_{7,64}=42.15$, $p<0.0001$). **(b)** When administered 15 min before testing (4 males – 4 females per genotype and treatment), the optimal dose of 0.3 IU OT restored the number of nose contacts in *Oprm1* null mice ($H_{3,32}=23.67$, $p<0.0001$), partially restored the time spent in nose contact ($H_{3,32}=28.28$, $p<0.0001$) but failed to normalize the time spent in ($H_{3,32}=27.31$, $p<0.0001$) and number of paw contacts ($H_{3,32}=27.27$, $p<0.0001$) and following episodes ($H_{3,32}=25.89$, $p<0.0001$). However, OT reduced the number of grooming episodes ($H_{3,32}=11.60$, $p<0.01$) and grooming after social contact in mutant mice ($H_{3,32}=22.27$, $p=0.0001$). **(c)** When administered 30 min before testing (4 males – 4 females per genotype and treatment), per nasal OT at 0.3 IU failed to relieve social interaction deficit in *Oprm1* null mice. **(d)** The non-peptide OT antagonist LIT183 or its vehicle (doses of 0, 7.5 or 15 mg/kg) were administered intraperitoneally 25 min before per nasal OT administration (0.3 IU) and 30 min before direct social interaction test (4 males – 4 females per genotype, LIT183 doses and OT treatment). *Oprm1* null mice treated with intranasal vehicle displayed reduced time spent in nose ($H_{11,94}=71.65$, $p<0.0001$) and paw contact ($H_{11,94}=49.21$, $p<0.0001$) that were not detected in *Oprm1* null mice treated with intranasal OT, except for the 15 mg/kg dose of LIT183 in the former. *Oprm1*$^{-/-}$ mice of the vehicle/vehicle group displayed a reduced number of following episodes ($H_{11,94}=32.92$, $p<0.001$), not significantly detected in other treatment groups. **(e)** We performed a modified version of the 3-chamber test (*Oprm1*$^{+/+}$ vehicle: 6 males – 7 females, *Oprm1*$^{+/+}$ OT: 7 males - 8 females, *Oprm1*$^{-/-}$ vehicle: 5 males - 8 females, *Oprm1*$^{-/-}$ OT: 6 males – 8 females). During the social preference phase, intranasal OT increased the time spent in (*Genotype [G] x Dose [D] x Stimulus [S]:* $F_{1,47}=76.4$, $p<0.0001$) and number (*G x D x S:* $F_{1,47}=26.19$, $p<0.0001$) of nose contacts with a stranger mouse versus a toy in *Oprm1* null mice. **(f)** During the modified social novelty preference phase, vehicle-treated *Oprm1*$^{+/+}$ mice spent more time in nose contact (*G x D x S:* $F_{1,47}=127.00$, $p<0.0001$, $p<0.0001$) and made more numerous nose contacts (*G x D x S:* $F_{1,47}=77.63$, $p<0.0001$) with a cage mate versus the stranger mouse, and intranasal OT reversed this preference. Vehicle-treated *Oprm1*$^{-/-}$ mice failed to display a preference during this phase; OT administration in these mutants led them to spend more time in nose contact and make more numerous nose contacts with the cage mate versus the stranger mouse. Results are shown as scatter plots and mean ± sem. Solid stars: significant difference with the vehicle-treated *Oprm1*$^{+/+}$ group, Tuckey's post-hoc test following a two-way ANOVA or 2-tailed t-test following a Kruskal-Wallis analysis of variance; one symbol: p<0.05, two symbols: p<0.01; three symbols: p<0.001. Letters: significant difference with vehicle-treated *Oprm1*$^{-/-}$ group (2-tailed t-test or Tukey's post-hoc test); (c): p<0.05, (b): p<0.01, (a): p<0.001. IU: International Units, OT: oxytocin.

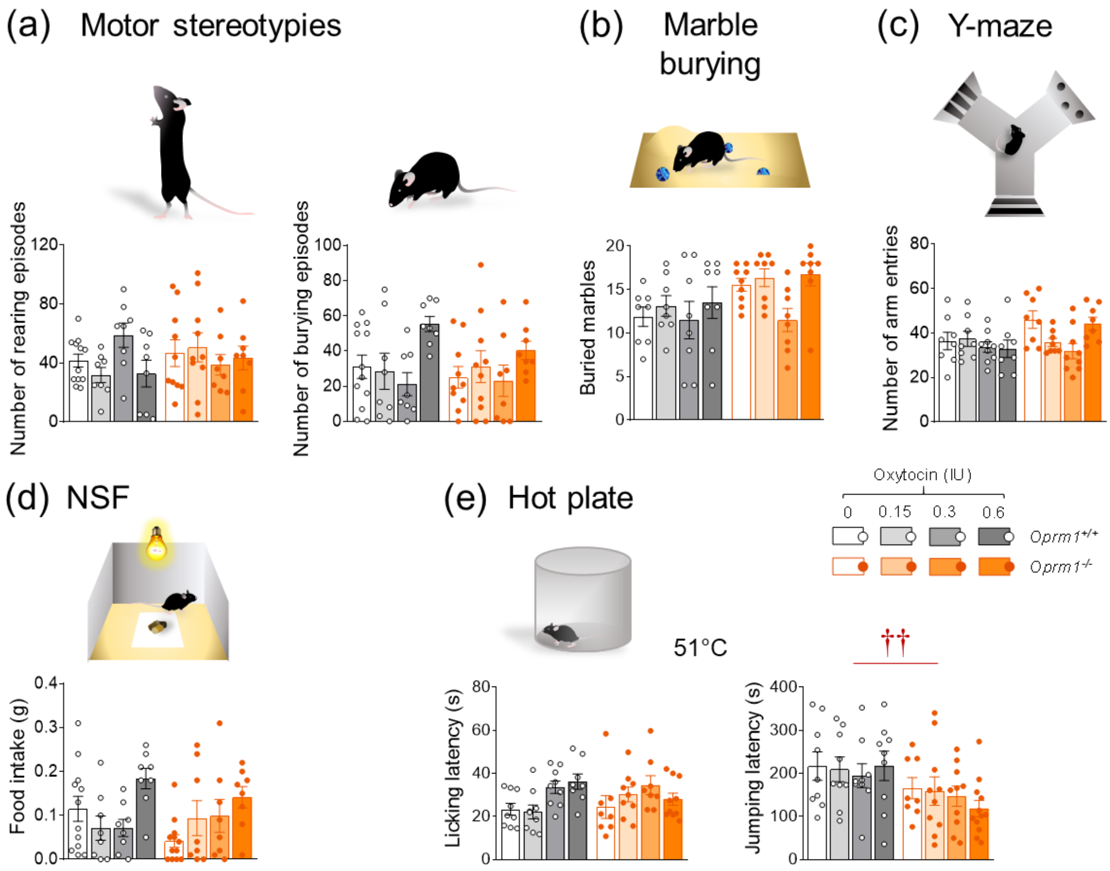

Figure S3. Acute per nasal OT relieved anxiety and induced analgesic effects in *Oprm1* null mice but had limited effects on stereotypies and perseveration. (a) When administered acutely 5 min before monitoring spontaneous motor stereotypies (*Oprm1*$^{+/+}$ vehicle: 6 males – 6 females, *Oprm1*$^{-/-}$ vehicle: 5 males - 5 females, *Oprm1*$^{-/-}$ OT 0.15 IU: 4 males – 6 females, other groups: 4 males – 4 females per genotype and dose), per nasal OT had no effect on rearing and burying behaviour in mutant and wild-type mice, suggesting no effect on general activity. **(b)** No significant effect of genotype nor OT administration was detected in the marble burying test (*Oprm1*$^{+/+}$ OT 0.6 IU: 5 males – 3 females, *Oprm1*$^{-/-}$ vehicle: 5 males - 4 females, other groups: 4 males – 4 females per genotype and dose). **(c)** In the Y-maze (*Oprm1*$^{-/-}$ OT 0.3 and 0.6 IU: 4 males – 5 females, other groups: 4 males – 4 females per genotype and dose), neither genotype nor treatment modified the number of arm entries. **(d)** In the novelty-suppressed-feeding test (*Oprm1*$^{+/+}$ and *Oprm1*$^{-/-}$ vehicle: 6 males – 6 females, other groups: 4 males – 4 females per genotype and dose), food intake was not modified by genotype nor by treatment. **(e)** In the hot plate test (same mice as for tail immersion), neither genotype nor OT treatment had a significant influence on nociceptive thresholds. Results are shown as scatter plots and mean ± sem. Daggers: genotype effect; one symbol: $p<0.05$, two symbols: $p<0.01$; three symbols: $p<0.001$.

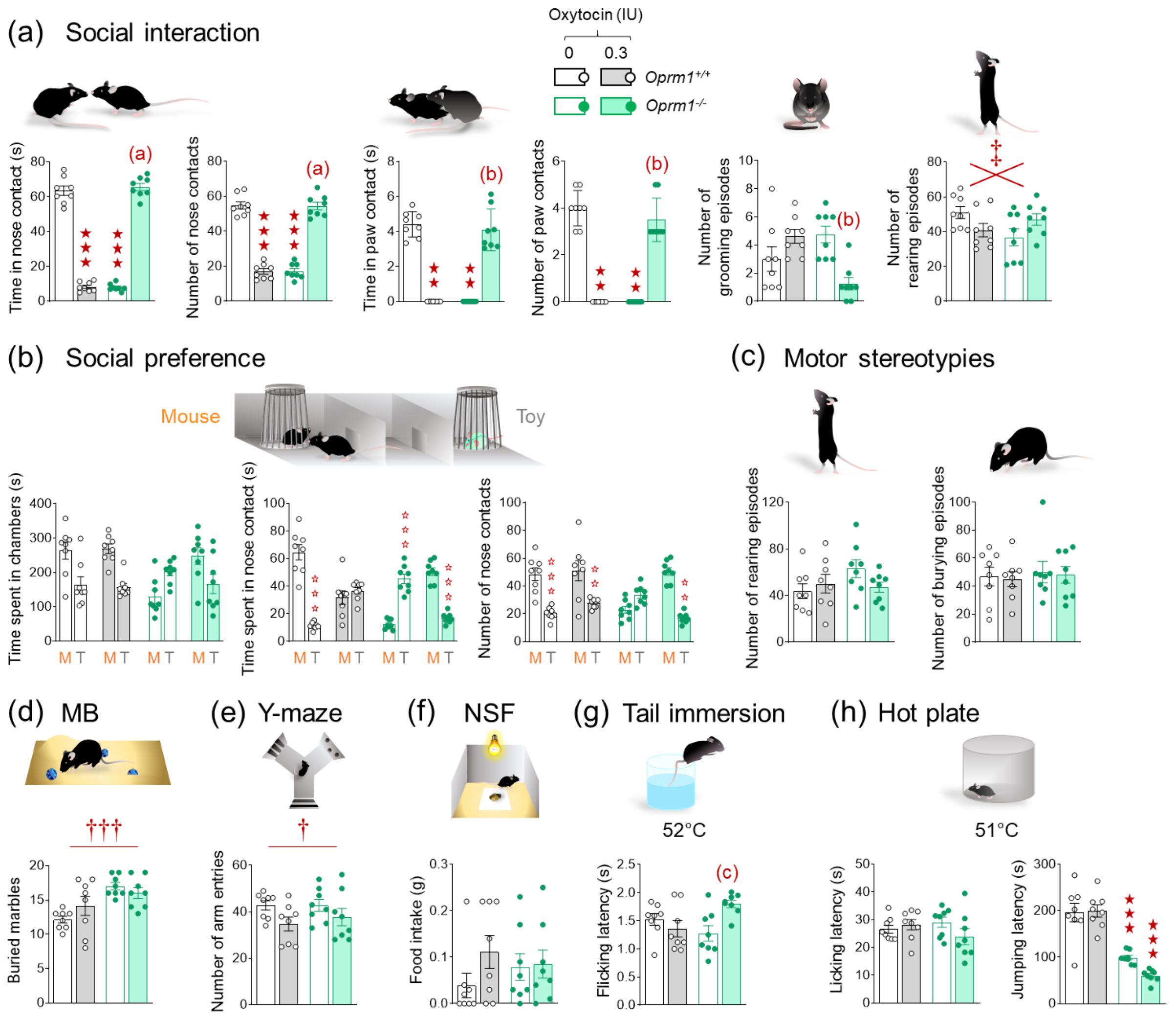

Figure S4. Chronic per nasal administration of OT at 0.3IU restored social interaction and suppressed stereotypies and anxiety-like behavior in *Oprm1* null mice but had deleterious effects in the same behaviors in their WT counterparts. *Oprm1*$^{+/+}$ and *Oprm1*$^{-/-}$ mice received 0.3IU of OT or vehicle (4 males – 4 females per genotype and treatment) once a day for 17 consecutive days, 5 min before testing (timeline in Figure 3a). **(a)** Vehicle-treated *Oprm1*$^{-/-}$ mice displayed a severe deficit in social interaction parameters; OT treatment fully reversed these deficits while producing severe impairment in *Oprm1*$^{+/+}$ controls (time spent in nose contact: *G x T:* $F_{1,28}=994.1$, $p<0.0001$; number of nose contacts: *G x T:* $F_{1,28}=405.8$; $p<0.0001$, time spent in paw contact: $H_{3,32}=27.0$, $p<0.0001$; number of paw contacts: $H_{3,32}=27.7$, $p<0.0001$; number of grooming episodes: $H_{3,32}=14.5$, $p<0.01$; number of rearing episodes: *G x T:* $F_{1,28}=6.8$; $p<0.05$). **(b)** In the social preference test, repeated OT exposure impaired preference for the mouse over the toy in *Oprm1*$^{+/+}$ mice, but rescued this preference in *Oprm1*$^{-/-}$ mice (time in nose contact: *G x T x S:* $F_{1,28}=165.7$, $p<0.0001$; number of nose contacts: *G x T x S:* $F_{1,28}=13.5$, $p<0.01$). **(c)** Genotype and treatment had no influence on the number of rearing and burying episodes when assessing spontaneous motor stereotypies. **(d)** In the marble burying test, increased marble burying was observed in OT-treated as well as in vehicle-treated *Oprm1* knockouts ($H_{3,32}=12.9$, $p<0.01$). **(e)** In the Y-maze test, *Oprm1*$^{-/-}$ mice made more arm entries *than* controls (*G:* $F_{1,28}=5.1$, $p<0.05$). **(f)** Neither genotype nor treatment influenced the amount of food consumed following the NSF test. **(g)** In the tail immersion test at 52°C, chronic OT increased the nociceptive threshold in *Oprm1* mutants ($H_{3,32}=8.2$, $p<0.05$).

(h) In the hot plate test, chronic OT failed to normalize lowered jumping latency in *Oprm1*$^{-/-}$ mice (*G:* $F_{1,28}=91.2$, $p<0.0001$). Results are shown as scatter plots and mean ± sem. Solid stars: significant difference with the vehicle-treated *Oprm1*$^{+/+}$ group, Tuckey's post-hoc test following a two-way ANOVA or 2-tailed t-test following a Kruskal-Wallis analysis of variance; open stars: genotype x treatment (Y-maze) or genotype x treatment x stimulus interaction (Social preference - stimulus: mouse/toy), Tukey's post-hoc test following an analysis of variance (ANOVA); daggers: genotype effect; one symbol: p<0.05, two symbols: p<0.01; three symbols: p<0.001. Letters: significant difference with vehicle-treated *Oprm1*$^{-/-}$ group (2-tailed t-test or Tukey's post-hoc test); (b): p<0.01, (a): p<0.001. IU: International Units, NSF: novelty-suppressed feeding test, MB: marble burying test.

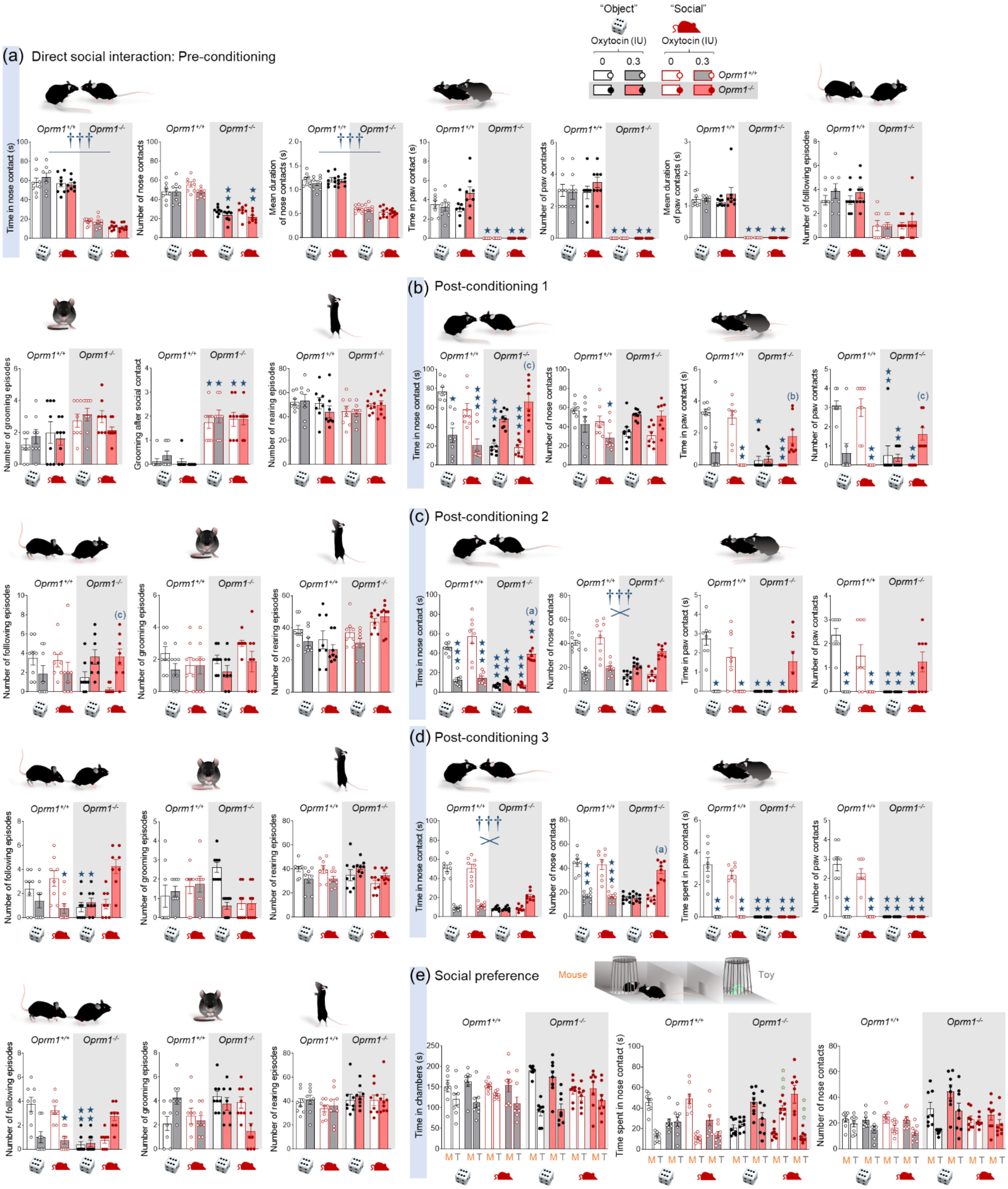

Figure S5. Beneficial effects of repeated intranasal OT on social deficit in *Oprm1* null mice were greater and lasted longer when associated with social experience. Social interaction experiments. After a pre-conditioning social interaction session, mice received per nasal OT (0.3 IU) or vehicle administration paired with the presentation of an unfamiliar object ("object" condition) or mouse ("social" condition) every two/three days over 2 weeks (D4

to D15) (4 males – 4 females per genotype, treatment and conditioning paradigm). A first post-conditioning social interaction session took place on D18, two days before 3-chamber test for social novelty preference (D20). Social interaction was assessed during two additional post-conditioning sessions, a week (D25) and two weeks (D32) after the first post-conditioning session (timeline in Figure 4a). **(a)** During the pre-conditioning social interaction session (no OT exposure yet), *Oprm1*$^{-/-}$ mice showed significant deficits in social behaviour compared to *Oprm1*$^{+/+}$ mice, as illustrated by decreased number of nose (*G:* $F_{1,56}$*=9481.89; p<0.0001*) and paw contacts ($H_{7,64}$*=55.36, p<0.0001*), time spent in nose ($H_{7,64}$*=50.38, p<0.0001*) and paw contacts ($H_{7,64}$*=55.42, p<0.0001*) and their duration (nose: *G:* $F_{1,56}$*=482.00; p<0.0001;* paw: $H_{7,64}$*=55.8, p<0.0001*), as well as a decreased number of following episodes ($H_{7,64}$*=37.04, p<0.0001*). In addition, *Oprm1*$^{-/-}$ mice showed an increased number of grooming episodes after a social contact ($H_{7,64}$*=37.4, p<0.0001*), but no difference in the total number of grooming episodes nor the number of rearing episodes. **(b)** During the first post-conditioning session, OT severely impaired social interaction in *Oprm1*$^{+/+}$ mice while restoring it in *Oprm1*$^{-/-}$ mice (time spent in nose contact: $H_{7,64}$*=41.4, p<0.0001*; number of nose contacts: $H_{7,64}$*=22.3, p<0.01*; time spent in paw contact: $H_{7,64}$*=42.6, p<0.0001;* number of paw contacts: $H_{7,64}$*=44.7, p<0.0001;* number of following episodes: $H_{7,64}$*=22.2, p<0.0001*). **(c)** During the second post-conditioning social interaction session (D25), social interaction remained severely impaired in OT-treated *Oprm1*$^{+/+}$ mice, while prosocial effects of OT could still be detected in OT-treated *Oprm1*$^{-/-}$ mice but only when tested under the social setting (time in nose contact: *G x T X P:* $F_{1,56}$*=15.6, p<0.001;* number of nose contacts: *G x T:* $F_{1,56}$*=108.5, p<0.0001;* time spent in paw contact: $H_{7,64}$*=45.9, p<0.0001*, number of paw contacts: $H_{7,64}$*=46.5, p<0.0001;* number of following episodes: $H_{7,64}$*=24.8, p<0.001*; grooming after social contact: $H_{7,64}$*=36.06, p<0.0001*). **(d)** After an additional week (D32), while a social interaction deficit was still observed in OT-treated *Oprm1*$^{+/+}$ mice, a few social parameters were still improved in OT-treated *Oprm1*$^{-/-}$ mice when tested under the “social” setting only (time spent in nose contact: *G x T:* $F_{1,56}$*=242.28, p<0.0001;* number of nose contacts: *G x T X P:* $F_{1,56}$*=10.9, p<0.01;* time spent in paw contact: $H_{7,64}$*=61.4, p<0.0001*; number of paw contacts: $H_{7,64}$*=61.6, p<0.0001;* number of following episodes: $H_{7,64}$*=38.5, p<0.00001*). **(e)** In the three-chamber test, time spent in nose contact with the mouse versus the object was fully restored in *Oprm1*$^{-/-}$ mice tested under the “social” but not “object” paradigm (*S x T x P:* $F_{1,28}$*=14.2, p<0.001).* Results are shown as scatter plots and mean ± sem. Solid stars: significant difference with the vehicle-treated Oprm1$^{+/+}$ group, Tuckey’s post-hoc test following a two-way ANOVA or 2-tailed t-test following a Kruskal-Wallis analysis of variance; open stars: genotype x treatment (Y-maze) or genotype x treatment x stimulus interaction (Social preference - stimulus: mouse/toy or stranger/cage mate comparison), Tukey’s post-hoc test following an analysis of variance (ANOVA); daggers: genotype x treatment interaction; one symbol: p<0.05, two symbols: p<0.01; three symbols: p<0.001. Letters: significant difference with vehicle-treated Oprm1$^{-/-}$ group (2-tailed t-test or Tukey’s post-hoc test); (c): p<0.05, (b): p<0.01, (a): p<0.001. More behavioural parameters in Fig. S4. D: day, M: mouse, T: toy.

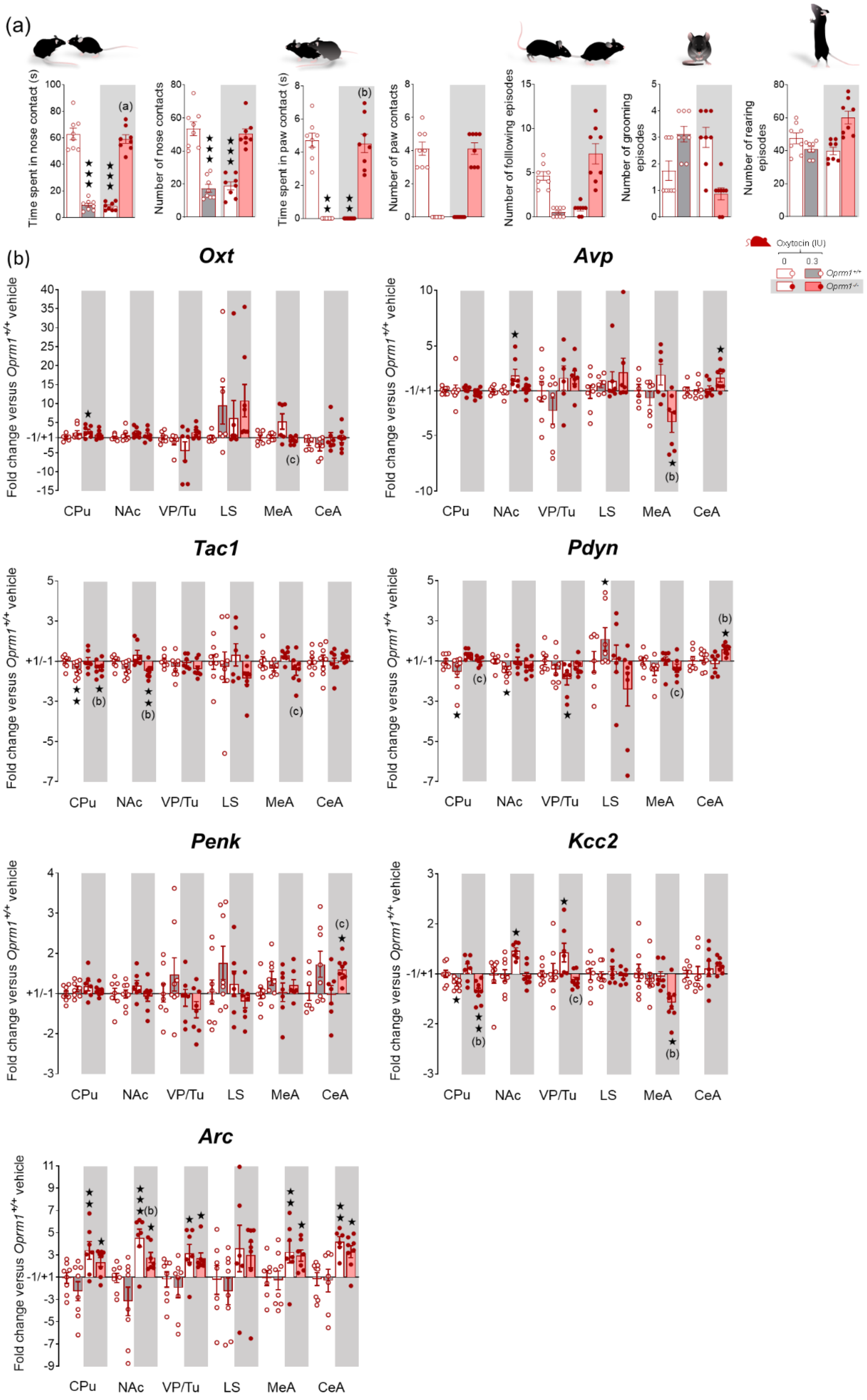
(a)
Time spent in nose contact (s)
Number of nose contacts
Time spent in paw contact (s)
Number of paw contacts
Number of folllowing episodes
Number of grooming episodes
Number of rearing episodes
Oxytocin (IU)
0
0.3
Oprm1+/+
Oprm1-/-
(b)
Oxt
Avp
Tac1
Pdyn
Penk
Kcc2
Arc
Fold change versus Oprm1+/+ vehicle
CPu
NAc
VP/Tu
LS
MeA
CeA

Figure S6. Transcriptional consequences of social OT conditioning in *Oprm1* null mice and their wild-type controls. See timeline in Figure 5. **(a)** OT exposure decreased social interaction in *Oprm1*$^{+/+}$ mice while rescuing it in *Oprm1*$^{-/-}$ mice (time spent in nose contact: $H_{3,32}=23.3$, $p<0.0001$; number of nose contacts: $F_{1,28}=119.3$, $p<0.0001$; time spent in paw contact: ($H_{3,32}=26.6$, $p<0.0001$); number paw contacts ($H_{3,32}=27.0$, $p<0.0001$), following episodes ($H_{3,32}=24.9$, $p<0.0001$) and grooming episodes ($H_{3,32}=16.9$, $p<0.001$). Per nasal OT increased the number of rearing episodes in *Oprm1*$^{-/-}$ mice ($F_{1,28}=21.9$, $p<0.0001$). **(b)** Genotype and OT treatment had little impact on the expression of oxytocin and vasopressin genes. Expression of *Kcc2* was increased in the Nac and VP/Tu of *Oprm1* null mice; OT decreased this expression in the CPu of *Oprm1*$^{+/+}$ and *Oprm1*$^{-/-}$ mice and in the MeA of *Oprm1*$^{-/-}$ mice. This treatment reduced *Kcc2* expression in the VP/Tu of *Oprm1*$^{-/-}$ mice when compared to vehicle-treated mutant mice. OT treatment reduced *Tac1* expression in the CPu of *Oprm1*$^{+/+}$ mice, and in the CPu, NAc and MeA (compared to vehicle-treated *Oprm1* null mice) of *Oprm1*$^{-/-}$ mice. OT administration decreased *Pdyn* expression in the CPu and NAc but increased it in the LS of *Oprm1*$^{+/+}$ mice; this expression was found decreased in the VP/Tu of vehicle-treated *Oprm1*$^{-/-}$ mice and in the MeA of OT-treated *Oprm1*$^{-/-}$ mice (compared to vehicle-treated *Oprm1* null mice) but increased in the CeA of OT-treated *Oprm1*$^{-/-}$ mice. Genotype and OT treatment had little impact on *Penk* expression. The expression of the immediate early gene *Arc* was found upregulated in the CPu, NAc, VP/Tu, MeA and CeA of *Oprm1*$^{-/-}$ mice; OT treatment reduced this expression in the NAc. Gene expression data are expressed as fold change versus *Oprm1*$^{+/+}$ - vehicle group (clustering or scatter plots and mean ± SEM). Comparison to *Oprm1*$^{+/+}$ - vehicle group (two-tailed t-test): One star p<0.05, two stars p<0.01, three stars p<0.001. Letters: significant difference with vehicle-treated *Oprm1*$^{-/-}$ group (2-tailed t-test); (c): p<0.05, (b): p<0.01, (a): p<0.001. qRT-PCR data are displayed in Table S2.

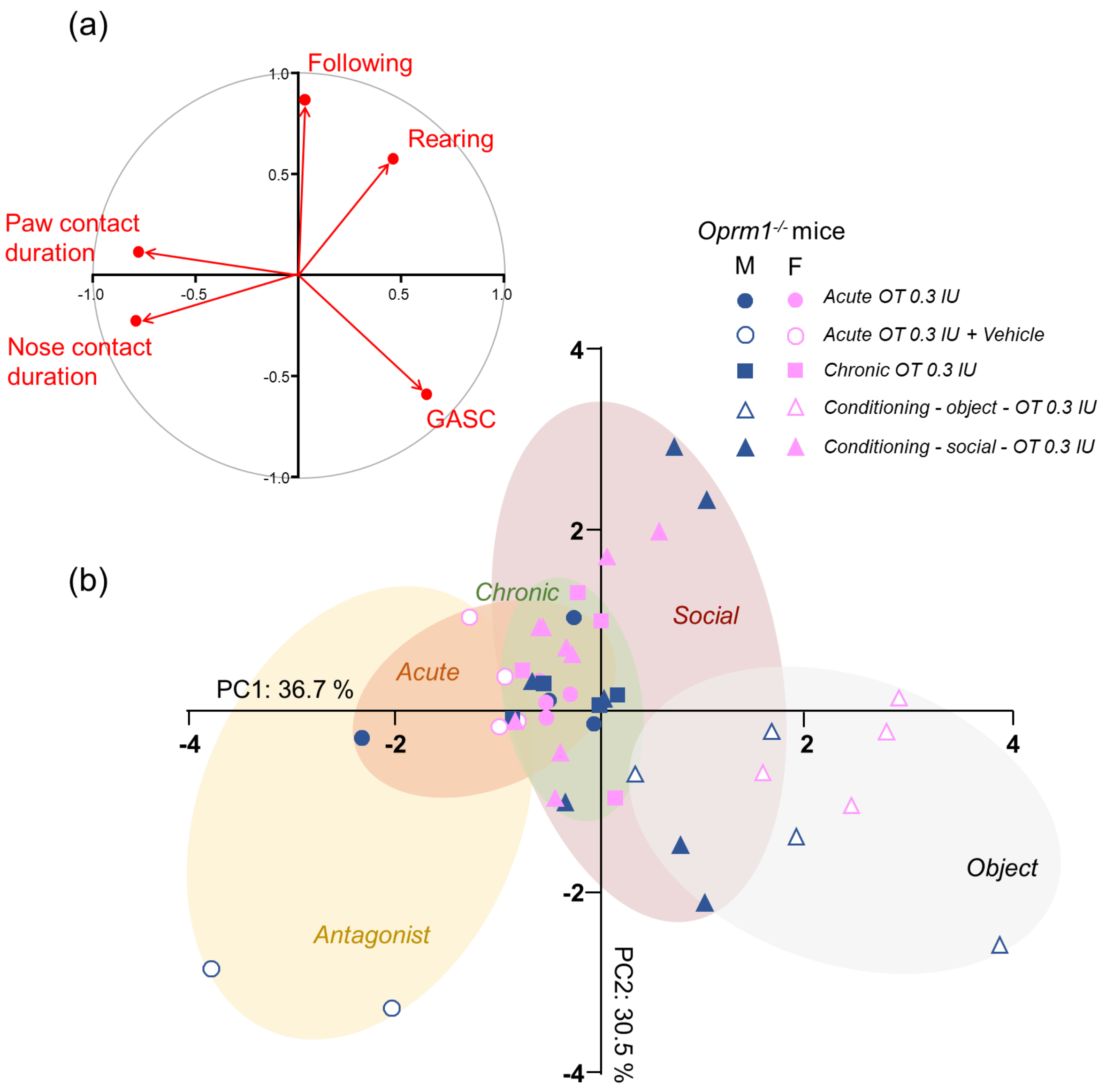

Figure S7. Principal component analysis (PCA) of social interaction parameters in *Oprm1* null mice treated with OT 0.3 IU. To assess whether sex influenced the effects of OT administration in *Oprm1* null mice, we performed a PCA on major social interaction parameters across the different experimental paradigms used in the study. Only the dose of 0.3 IU of OT and the 5 min delay after injection were considered, as common across experimental paradigms. Different paradigms were acute administration (5 min delay, Figure Aa), acute administration – effect of LIT183 (vehicle group, Figure 1d), chronic administration (Figure 3a), conditioning – object paradigm (Figure 4a), conditioning – social paradigm (Figures 4a and 5b). **(a)** PCA segregated social interaction in the factor space along two principal components (PCs). PC1 opposed pro-social parameters (nose and paw contact durations) to grooming after social contact, a sign of social discomfort. PC2 was mainly under the influence of locomotor activity, driven by the number of following episodes and rearing activity. **(b)** In subject space, male and female individuals were evenly distributed, suggesting that sex had no major influence on social behaviour in *Oprm1* null mice treated with OT under these different conditions. In contrast, *Oprm1* knockout individuals clustered by experimental paradigms,

showing notably that the "social" conditioning paradigm was more efficient to normalize behaviour along PC1 than the "object" paradigm.

References

1 Becker JA, Clesse D, Spiegelhalter C, Schwab Y, Le Merrer J, Kieffer BL. Autistic-like syndrome in mu opioid receptor null mice is relieved by facilitated mGluR4 activity. Neuropsychopharmacology. 2014;39(9):2049-60.

2 Pujol CN, Pellissier LP, Clement C, Becker JAJ, Le Merrer J. Back-translating behavioral intervention for autism spectrum disorders to mice with blunted reward restores social abilities. Transl Psychiatry. 2018;8(1):197.

3 Katayama Y, Nishiyama M, Shoji H, Ohkawa Y, Kawamura A, Sato T, et al. CHD8 haploinsufficiency results in autistic-like phenotypes in mice. Nature. 2016;537(7622):675-79.

4 Matsuo N, Tanda K, Nakanishi K, Yamasaki N, Toyama K, Takao K, et al. Comprehensive behavioral phenotyping of ryanodine receptor type 3 (RyR3) knockout mice: decreased social contact duration in two social interaction tests. Front Behav Neurosci. 2009;3:3.

5 Derieux C, Leaute A, Brugoux A, Jaccaz D, Terrier C, Pin JP, et al. Chronic sodium bromide treatment relieves autistic-like behavioral deficits in three mouse models of autism. Neuropsychopharmacology. 2022;47(9):1680-92.

6 Fonteneau M, Brugoux A, Jaccaz D, Donello JE, Banerjee P, Le Merrer J, et al. The NMDA receptor modulator zelquistinel durably relieves behavioral deficits in three mouse models of autism spectrum disorder. Neuropharmacology. 2024;248:109889.

7 Silverman JL, Yang M, Lord C, Crawley JN. Behavioural phenotyping assays for mouse models of autism. Nat Rev Neurosci. 2010;11(7):490-502.

8 Moy SS, Riddick NV, Nikolova VD, Teng BL, Agster KL, Nonneman RJ, et al. Repetitive behavior profile and supersensitivity to amphetamine in the C58/J mouse model of autism. Behav Brain Res. 2014;259:200-14.

9 Thomas A, Burant A, Bui N, Graham D, Yuva-Paylor LA, Paylor R. Marble burying reflects a repetitive and perseverative behavior more than novelty-induced anxiety. Psychopharmacology (Berl). 2009;204(2):361-73.

10 Delotterie D, Ruiz G, Brocard J, Schweitzer A, Roucard C, Roche Y, et al. Chronic administration of atypical antipsychotics improves behavioral and synaptic defects of STOP null mice. Psychopharmacology (Berl). 2010;208(1):131-41.

11 Le Marec N, Ethier K, Rompre PP, Godbout R. Involvement of the medial prefrontal cortex in two alternation tasks using different environments. Brain Cogn. 2002;48(2-3):432-6.

12 Zhou M, Rebholz H, Brocia C, Warner-Schmidt JL, Fienberg AA, Nairn AC, et al. Forebrain overexpression of CK1delta leads to down-regulation of dopamine receptors and altered locomotor activity reminiscent of ADHD. Proc Natl Acad Sci U S A. 2010;107(9):4401-6.

13 Becker JAJ, Pellissier LP, Corde Y, Laboute T, Leaute A, Gandia J, et al. Facilitating mGluR4 activity reverses the long-term deleterious consequences of chronic morphine exposure in male mice. Neuropsychopharmacology. 2021;46(7):1373-85.

14 Laurent P, Becker JA, Valverde O, Ledent C, de Kerchove d'Exaerde A, Schiffmann SN, et al. The prolactin-releasing peptide antagonizes the opioid system through its receptor GPR10. Nat Neurosci. 2005;8(12):1735-41.

15 Frantz MC, Pellissier LP, Pflimlin E, Loison S, Gandia J, Marsol C, et al. LIT-001, the First Nonpeptide Oxytocin Receptor Agonist that Improves Social Interaction in a Mouse Model of Autism. J Med Chem. 2018;61(19):8670-92.

16 Rae M, Gomes I, Spelta LEW, Bailey A, Marcourakis T, Devi L, et al. Environmental enrichment enhances ethanol preference over social reward in male swiss mice: Involvement of oxytocin-dopamine interactions. Neuropharmacology. 2024;253:109971.

Table S1. List of primers used for qRT-PCR

Refseq	Gene name	Gen title	Forward oligonucleotide	Reverse oligonucleotide
NM_009630	adenosine A2a receptor	*Adora2a*	TCAGCCTCTTGGCTATTGCC	CTCAAACAGACAGGTCACCCG
NM_009630	activity regulated cytoskeletal-associated protein	*Arc*	CCAGGAGAATGACACCAG	TTCAGGAGAAGAGAGGATG
NM_009732	arginine vasopressine	*Avp*	ACACTACGCTCTTCCGCTTGT	CACTGTCTCAGCTCCATGTCA
NM_016847	arginine vasopressine receptor 1A	*Avpr1a*	GGAGAAACGGGAGACAGACA	AAGCCCATTGTACAGCCCAAG
NM_011924	arginine vasopressine receptor 1B	*Avpr1b*	CTGCCTTCAGGTTCTTGCCT	TAATTCACAGGTCATGCGCCA
NM_205769	corticotropin releasing hormone	*Crh*	AGGAGGCATCCTGAGAGAAGT	ATGTTAGGGGCGCTCTCTTC
NM_010076	dopamine receptor D1A	*Drd1a*	AGATCGGGCATTTGGAGAG	GGATGCTGCCTCTTCTTCTG
NM_010077	dopamine receptor D2	*Drd2*	TGCCATTGTTCTTGGTGTGT	GTGAAGGCGCTGTAGAGGAC
NM_010234	FBJ osteosarcoma oncogene	*Fos*	GAAGGGAACGGAATAAGATG	CATCTTCAAGTTGATCTGTCTC
NM_001160353	glutamate receptor, metabotropic 2	*Grm2*	CTTGTAGCTATGCCCCGTGT	GACTGGAAGCACCTTTGCAT
NM_001013385	glutamate receptor, metabotropic 4	*Grm4*	CTTTCTCTGCTATGCCACCACC	TAGCTGATGCTCATGCCAAGCC
NM_011025	oxytocin	*Oxt*	CTGCTTGGCTTACTGGCTCT	GGGAGACACTTGCGCATATC
NM_001081147	oxytocin receptor	*Oxtr*	CTTAGGGCCAAAAGGTGTCA	GCAGGTTTCTATGCCCTCTG
NM_018863	prodynorphin	*Pdyn*	TTTGGCAACGGAAAAGAATC	TAGCGTTTGGCCTGTTTTCT
NM_001002927	preproenkephalin	*Penk*	ATGCAGATGAGGGAGACACC	GCTTCTGCAGCTCTTTTGCT
NM_009194	solute carrier family 12, member 2	*Slc12a2*	AGGTAAAACATCCGGTGGGT	AGCACAAAGAGAAAGACGCAAC
NM_020333	solute carrier family 12, member 5	*Slc12a5*	CAGACCTATGTGCAGGGCAA	CCGAGTCGGGATGCGAAATA
NM_080853	solute carrier family 17 (sodium-dependent inorganic phosphate cotransporter), member 6	*Slc17a6*	AGGCCCTGCTACTGCAAATA	GACACAAAGCAGAGAGGGACT
NM_182993	solute carrier family 17 (sodium-dependent inorganic phosphate cotransporter), member 7	*Slc17a7*	GGCCATTTGTTGTGTGTCCC	CTCACCCCCACCCAGATTTC
NM_009311	tachykinin 1	*Tac1*	CCGTTCACTGCTCACTGACACAG	CTCGTTTCCACTCAACTGTTTGC

Table S2: Transcription levels of a set of 19 genes in the CPu, NAc, VP/Tu, CeA, MeA, VP and LS in Oprm1+/+ or Oprm1-/- mice treated chronically with OT versus vehicle

			NAc-Oprm1$^{-/-}$/Vehicle				NAc-Oprm1$^{+/+}$/OXT				NAc-Oprm1$^{-/-}$/OXT			
Refseq	Gene name	Gen title	FC	sem	p values	Benjamini-Hochberg Adjusted P value	FC	sem	p values	Benjamini-Hochberg Adjusted P value	FC	sem	p values	Benjamini-Hochberg Adjusted P value
NM_009630	adenosine A2a receptor	*Adora2a*	-1.17	0.69	0.196	0.727	**-1.94**	**0.21**	**0.009**	0.399	**-2.09**	**0.22**	**0.004**	0.090
NM_009630	activity regulated cytoskeletal-associated protein	*Arc*	**4.52**	**0.19**	**0.238**	**0.025**	-3.19	1.28	0.139	0.726	**2.75**	**0.42**	**0.010**	0.090
NM_009732	arginine vasopressine	*Avp*	**2.37**	**0.79**	**0.001**	0.197	-1.15	0.15	0.473	0.618	1.18	0.19	0.488	0.090
NM_016847	arginine vasopressine receptor 1A	*Avpr1a*	-1.12	0.28	0.701	0.904	-1.69	0.49	0.242	0.227	1.43	0.39	0.437	0.094
NM_011924	arginine vasopressine receptor 1B	*Avpr1b*	1.24	0.35	0.145	0.347	**-1.49**	**0.18**	**0.032**	0.726	-1.04	0.09	0.708	0.094
NM_205769	corticotropin releasing hormone	*Crh*	-1.08	0.49	0.052	0.923	1.18	0.47	0.694	0.105	1.37	0.26	0.300	0.309
NM_010076	dopamine receptor D1A	*Drd1a*	1.12	0.16	0.825	0.667	**-1.61**	**0.15**	**0.007**	0.820	**-1.47**	**0.13**	**0.020**	0.309
NM_010077	dopamine receptor D2	*Drd2*	1.33	0.57	0.812	0.384	-1.13	0.10	0.718	0.726	-1.44	0.12	0.134	0.309
NM_010234	FBJ osteosarcoma oncogene	*Fos*	1.49	0.09	0.105	0.421	-1.25	0.21	0.465	0.820	-1.01	0.24	0.917	0.309
NM_00116035	glutamate receptor, metabotropic 2	*Grm2*	**-1.06**	**0.55**	**0.032**	0.904	-1.06	0.19	0.727	0.354	-1.21	0.16	0.269	0.535
NM_00101338	glutamate receptor, metabotropic 4	*Grm4*	**-1.39**	**0.05**	**0.013**	0.197	-1.43	0.22	0.107	0.726	**-1.68**	**0.22**	**0.017**	0.535
NM_011025	oxytocin	*Oxt*	2.25	0.06	0.080	0.241	1.59	0.56	0.411	0.721	1.64	0.54	0.360	0.535
NM_00108114	oxytocin receptor	*Oxtr*	1.04	0.16	0.712	0.904	-1.32	0.28	0.333	0.227	-1.24	0.17	0.314	0.535
NM_018863	prodynorphin	*Pdyn*	**1.19**	**0.11**	**0.034**	0.472	-1.00	0.1	0.989	0.820	-1.07	0.12	0.743	0.535
NM_00100292	preproenkephalin	*Penk*	1.00	0.32	0.888	0.923	**-1.43**	**0.14**	**0.049**	0.989	-1.34	0.13	0.107	0.535
NM_009194	solute carrier family 12, member 2	*Slc12a2*	-1.17	0.12	0.287	0.306	-1.18	0.18	0.344	0.989	-1.22	0.11	0.129	0.624
NM_020333	solute carrier family 12, member 5	*Slc12a5*	1.45	0.18	0.923	0.157	-1.03	0.11	0.986	0.820	-1.04	0.07	0.938	0.854
NM_080853	solute carrier family 17 (sodium-dependent inorganic phosphate cotransporter), member 6	*Slc17a6*	-1.19	0.22	0.200	0.904	-1.40	0.73	0.679	0.354	1.03	0.52	0.912	0.854
NM_182993	solute carrier family 17 (sodium-dependent inorganic phosphate cotransporter), member 7	*Slc17a7*	1.77	0.09	0.435	0.384	-1.56	0.18	0.097	0.820	-1.26	0.17	0.372	0.938
NM_009311	tachykinin 1	*Tac1*	1.31	0.13	0.172	0.384	**-1.40**	**0.13**	**0.042**	0.227	**-1.48**	**0.12**	**0.011**	0.938

	CPu-Oprm1$^{-/-}$/Vehicle				CPu-Oprm1$^{+/+}$/OXT				CPu-Oprm1$^{-/-}$/OXT			
Gen title	FC	sem	p values	Benjamini-Hochberg Adjusted P value	FC	sem	p values	Benjamini-Hochberg Adjusted P value	FC	sem	p values	Benjamini-Hochberg Adjusted P value
Adora2a	-1.01	0.13	0.977	0.977	**-1.63**	**0.08**	**0.000**	**0.001**	**-1.64**	**0.09**	**0.000**	**0.000**
Arc	**3.86**	**0.70**	**0.005**	0.071	-2.26	0.86	0.249	0.393	**2.35**	**0.41**	**0.041**	0.080
Avp	1.07	0.14	0.713	0.887	1.05	0.46	0.912	0.912	-1.25	0.13	0.170	0.293
Avpr1a	1.72	0.24	0.167	0.462	**-1.96**	**0.19**	**0.030**	0.089	**-2.40**	**0.51**	**0.032**	0.068
Avpr1b	-1.30	0.26	0.306	0.703	**-1.72**	**0.18**	**0.006**	0.056	**-2.21**	**0.34**	**0.005**	**0.022**
Crh	1.08	0.28	0.886	0.970	1.07	0.07	0.840	0.904	-1.01	0.31	0.934	0.934
Drd1a	1.04	0.07	0.628	0.887	**-1.26**	**0.08**	**0.017**	0.080	**-1.38**	**0.04**	**0.000**	**0.000**
Drd2	-1.04	0.10	0.733	0.887	**-1.25**	**0.08**	**0.045**	0.115	**-1.59**	**0.06**	**0.000**	**0.000**
Fos	1.51	0.24	0.144	0.462	-1.60	0.28	0.110	0.211	-1.27	0.13	0.243	0.350
Grm2	1.09	0.09	0.713	0.887	-1.10	0.11	0.813	0.904	-1.59	0.30	0.206	0.316
Grm4	1.05	0.12	0.821	0.944	-1.23	0.22	0.363	0.491	**-1.43**	**0.13**	**0.030**	0.068
Oxt	**2.61**	**0.55**	**0.024**	0.142	2.24	0.61	0.107	0.211	1.72	0.39	0.178	0.293
Oxtr	1.21	0.14	0.481	0.790	-1.28	0.16	0.235	0.393	**-1.66**	**0.20**	**0.028**	0.068
Pdyn	1.18	0.12	0.180	0.462	1.11	0.06	0.256	0.393	1.07	0.04	0.394	0.453
Penk	**1.33**	**0.08**	**0.044**	0.206	-1.53	0.28	0.110	0.211	1.01	0.07	0.932	0.934
Slc12a2	**-1.35**	**0.08**	**0.008**	0.071	**-1.37**	**0.09**	**0.009**	0.056	**-1.71**	**0.14**	**0.000**	**0.003**
Slc12a5	1.10	0.09	0.374	0.717	**-1.20**	**0.03**	**0.025**	0.089	**-1.35**	**0.07**	**0.003**	**0.018**
Slc17a6	**2.14**	**0.29**	**0.009**	0.071	-1.25	0.39	0.600	0.767	1.597	0.45	0.287	0.367
Slc17a7	1.56	0.64	0.427	0.756	**-2.93**	**0.65**	**0.031**	0.089	**-2.45**	**0.31**	**0.009**	**0.029**
Tac1	1.16	0.09	0.102	0.391	**-1.23**	**0.06**	**0.007**	0.056	**-1.30**	**0.09**	**0.011**	**0.032**

	CeA-Oprm1$^{-/-}$/Vehicle				CeA-Oprm1$^{+/+}$/OXT				CeA-Oprm1$^{-/-}$/OXT			
Gen title	FC	sem	p values	Benjamini-Hochberg Adjusted P value	FC	sem	p values	Benjamini-Hochberg Adjusted P value	FC	sem	p values	Benjamini-Hochberg Adjusted P value
Adora2a	-1.28	0.15	0.503	0.925	-1.51	0.31	0.296	0.858	1.05	0.17	0.824	0.824
Arc	**4.18**	**0.42**	**0.001**	**0.023**	-1.30	0.96	0.912	0.983	**3.29**	**0.53**	**0.007**	0.159
Avp	1.18	0.26	0.712	0.925	1.12	0.28	0.831	0.983	**2.15**	**0.36**	**0.039**	0.824
Avpr1a	1.08	0.16	0.865	0.925	-1.15	0.29	0.601	0.983	1.12	0.17	0.775	0.418
Avpr1b	-1.14	0.16	0.497	0.925	**-1.57**	**0.21**	**0.038**	0.296	-1.2	0.08	0.236	0.824
Crh	-1.10	0.13	0.809	0.925	1.60	0.25	0.203	0.858	1.69	0.27	0.154	0.530
Drd1a	1.05	0.13	0.859	0.925	-1.21	0.20	0.511	0.983	1.23	0.05	0.368	0.748
Drd2	-1.38	0.26	0.225	0.925	1.03	0.33	0.983	0.983	1.13	0.13	0.650	0.414
Fos	1.47	0.23	0.081	0.624	1.19	0.18	0.373	0.858	1.20	0.11	0.216	0.685
Grm2	**-1.54**	**0.20**	**0.038**	0.446	-1.24	0.16	0.259	0.858	1.14	0.18	0.547	0.450
Grm4	-1.46	0.24	0.125	0.720	1.03	0.41	0.933	0.983	1.28	0.20	0.283	0.685
Oxt	-1.02	0.42	0.931	0.931	-1.70	0.47	0.298	0.858	1.51	0.63	0.566	0.340
Oxtr	-1.33	0.33	0.322	0.925	**-1.44**	**0.15**	**0.031**	0.296	-1.20	0.10	0.135	0.110
Pdyn	-1.03	0.23	0.866	0.925	1.72	0.32	0.092	0.530	**1.59**	**0.10**	**0.016**	0.340
Penk	-1.15	0.20	0.533	0.925	1.06	0.16	0.879	0.983	**1.58**	**0.09**	**0.019**	0.110
Slc12a2	-1.04	0.18	0.805	0.925	1.06	0.19	0.831	0.983	1.18	0.21	0.528	0.685
Slc12a5	1.10	0.15	0.548	0.925	1.00	0.14	0.966	0.983	1.16	0.05	0.082	0.243
Slc17a6	1.14	0.31	0.822	0.925	1.01	0.10	0.731	0.983	**-1.76**	**0.29**	**0.017**	0.110
Slc17a7	-1.08	0.29	0.754	0.925	**-1.60**	**0.22**	**0.020**	0.296	-1.30	0.15	0.084	0.243
Tac1	-1.10	0.24	0.706	0.925	1.03	0.27	0.948	0.983	1.20	0.06	0.293	0.450

	LS-Oprm1$^{-/-}$/Vehicle				LS-Oprm1$^{+/+}$/OXT				LS-Oprm1$^{-/-}$/OXT			
Gen title	FC	sem	p values	Benjamini-Hochberg Adjusted P value	FC	sem	p values	Benjamini-Hochberg Adjusted P value	FC	sem	p values	Benjamini-Hochberg Adjusted P value
Adora2a	-1.36	0.75	0.641	0.827	1.44	1.37	0.904	0.951	-2.21	0.84	0.290	0.574
Arc	3.58	1.61	0.260	0.827	-2.26	1.07	0.575	0.910	3.00	1.03	0.218	0.558
Avp	1.86	0.70	0.351	0.827	1.59	0.24	0.361	0.910	2.676	1.04	0.200	0.558
Avpr1a	1.62	0.25	0.222	0.827	1.22	0.24	0.626	0.910	1.26	0.10	0.494	0.617
Avpr1b	-1.28	0.24	0.442	0.827	-1.03	0.42	0.951	0.951	**-2.49**	**0.57**	**0.049**	0.424
Crh	1.21	0.44	0.640	0.827	2.03	0.48	0.091	0.770	1.66	0.63	0.359	0.574
Drd1a	1.69	0.42	0.625	0.827	2.05	0.33	0.196	0.770	-1.02	0.31	0.350	0.574
Drd2	-1.36	0.12	0.091	0.827	1.10	0.13	0.598	0.910	-1.19	0.15	0.399	0.574
Fos	1.22	0.61	0.802	0.827	1.65	1.04	0.628	0.910	-1.97	0.51	0.332	0.574
Grm2	1.23	0.09	0.152	0.898	-1.04	0.16	0.833	0.951	1.15	0.03	0.136	0.558
Grm4	1.07	0.07	0.551	0.827	1.22	0.13	0.225	0.770	-1.03	0.08	0.862	0.862
Oxt	6.16	3.87	0.295	0.827	9.46	4.08	0.113	0.770	10.75	3.80	0.055	0.424
Oxtr	1.14	0.06	0.393	0.827	-1.02	0.10	0.927	0.951	1.18	0.05	0.252	0.574
Pdyn	1.24	0.26	0.647	0.827	1.76	0.36	0.178	0.770	-1.19	0.14	0.509	0.617
Penk	1.10	0.56	0.859	0.898	2.08	0.47	0.170	0.770	-2.41	0.73	0.200	0.558
Slc12a2	1.00	0.11	0.978	0.827	-1.15	0.24	0.633	0.910	-1.22	0.15	0.397	0.574
Slc12a5	1.05	0.07	0.683	0.827	-1.02	0.06	0.905	0.951	-1.04	0.04	0.779	0.814
Slc17a6	1.08	0.34	0.853	0.978	1.20	0.22	0.539	0.910	1.27	0.30	0.504	0.617
Slc17a7	-1.18	0.21	0.620	0.827	-1.21	0.18	0.522	0.910	-1.19	0.43	0.761	0.814
Tac1	1.32	0.43	0.574	0.898	-1.29	0.67	0.774	0.951	-1.85	0.27	0.107	0.558

	MeA-Oprm1$^{-/-}$/Vehicle				MeA-Oprm1$^{+/+}$/OXT				MeA-Oprm1$^{-/-}$/OXT			
Gen title	FC	sem	p values	Benjamini-Hochberg Adjusted P value	FC	sem	p values	Benjamini-Hochberg Adjusted P value	FC	sem	p values	Benjamini-Hochberg Adjusted P value
Adora2a	-1.13	0.15	0.642	0.896	**-1.66**	**0.10**	**0.045**	0.259	**-2.10**	**0.36**	**0.028**	0.066
Arc	**4.08**	**0.64**	**0.008**	0.093	-1.16	0.87	0.938	0.938	**2.93**	**0.47**	**0.034**	0.072
Avp	2.44	0.85	0.218	0.750	-1.67	0.58	0.409	0.587	**-3.80**	**0.86**	**0.027**	0.066
Avpr1a	1.12	0.16	0.540	0.887	**-1.77**	**0.11**	**0.000**	**0.007**	**-1.88**	**0.14**	**0.000**	**0.006**
Avpr1b	-1.21	0.16	0.437	0.839	**-1.59**	**0.17**	**0.041**	0.259	-1.36	0.18	0.204	0.247
Crh	-1.38	0.29	0.359	0.752	1.03	0.16	0.800	0.836	-1.48	0.18	0.147	0.212
Drd1a	-1.41	0.5	0.539	0.887	-1.61	0.25	0.201	0.385	-1.69	0.19	0.118	0.182
Drd2	-1.35	0.3	0.293	0.750	-1.30	0.16	0.149	0.358	**-2.22**	**0.26**	**0.001**	**0.009**
Fos	-1.05	0.24	0.803	0.939	-1.21	0.19	0.453	0.614	-1.35	0.26	0.316	0.339
Grm2	-1.39	0.27	0.284	0.750	-1.18	0.16	0.559	0.676	-1.44	0.13	0.095	0.156
Grm4	**-1.63**	**0.12**	**0.038**	0.294	-1.56	0.26	0.138	0.358	**-2.38**	**0.29**	**0.003**	**0.015**
Oxt	5.26	1.89	0.078	0.451	1.22	0.34	0.790	0.836	-1.70	0.33	0.345	0.345
Oxtr	1.16	0.25	0.596	0.896	-1.49	0.26	0.124	0.358	**-1.85**	**0.21**	**0.005**	**0.021**
Pdyn	1.03	0.23	0.921	0.963	1.38	0.17	0.107	0.358	1.20	0.13	0.325	0.339
Penk	-1.00	0.20	0.98	0.980	-1.50	0.22	0.089	0.358	-1.34	0.18	0.199	0.247
Slc12a2	-1.36	0.21	0.195	0.750	-1.25	0.16	0.302	0.464	**-1.81**	**0.13**	**0.001**	**0.010**
Slc12a5	-1.05	0.10	0.722	0.923	-1.11	0.13	0.543	0.676	**-1.57**	**0.12**	**0.015**	**0.050**
Slc17a6	1.01	0.13	0.857	0.939	-1.57	0.24	0.156	0.358	-1.58	0.10	0.054	0.104
Slc17a7	-1.17	0.45	0.835	0.939	-1.60	0.32	0.256	0.454	**-2.42**	**0.43**	**0.025**	0.066
Tac1	1.28	0.12	0.337	0.752	-1.35	0.12	0.171	0.358	-1.44	0.25	0.179	0.243

	VP-Oprm1$^{-/-}$/Vehicle				VP-Oprm1$^{+/+}$/OXT				VP-Oprm1$^{-/-}$/OXT			
Gen title	FC	sem	p values	Benjamini-Hochberg Adjusted P value	FC	sem	p values	Benjamini-Hochberg Adjusted P value	FC	sem	p values	Benjamini-Hochberg Adjusted P value
Adora2a	**-3.00**	**0.72**	**0.039**	0.168	-2.42	0.98	0.221	0.648	**-6.34**	**1.21**	**0.001**	**0.006**
Arc	**3.14**	**0.81**	**0.043**	0.168	-1.86	0.91	0.573	0.776	**2.69**	**0.45**	**0.044**	0.147
Avp	2.14	1.00	0.401	0.710	-2.81	1.08	0.248	0.648	2.19	0.53	0.271	0.389
Avpr1a	**1.65**	**0.24**	**0.015**	0.159	**-1.73**	**0.30**	**0.032**	0.648	**-1.57**	**0.12**	**0.000**	**0.006**
Avpr1b	1.05	0.11	0.821	0.908	-1.74	0.37	0.085	0.648	-1.05	0.19	0.842	0.881
Crh	-1.43	0.18	0.329	0.688	1.12	0.14	0.985	0.985	-1.32	0.15	0.383	0.464
Drd1a	-1.51	0.25	0.225	0.552	-1.48	0.29	0.282	0.648	**-2.62**	**0.37**	**0.005**	**0.020**
Drd2	-1.02	0.15	0.864	0.908	-1.16	0.17	0.469	0.756	**-2.04**	**0.21**	**0.001**	**0.006**
Fos	-1.04	0.21	0.868	0.908	-1.13	0.14	0.493	0.756	-1.19	0.03	0.201	0.331
Grm2	1.27	0.21	0.240	0.552	1.07	0.27	0.817	0.940	-1.05	0.04	0.452	0.520
Grm4	1.33	0.33	0.383	0.710	1.46	0.35	0.265	0.648	-1.26	0.15	0.183	0.331
Oxt	-4.62	2.49	0.146	0.420	-1.95	0.81	0.311	0.650	2.43	0.58	0.096	0.247
Oxtr	1.03	0.17	0.934	0.934	-1.48	0.25	0.109	0.648	**-1.86**	**0.17**	**0.001**	**0.006**
Pdyn	-1.11	0.21	0.706	0.903	1.47	0.41	0.341	0.654	-1.40	0.20	0.188	0.331
Penk	**-1.89**	**0.29**	**0.020**	0.159	-1.34	0.25	0.270	0.648	-1.29	0.14	0.245	0.376
Slc12a2	1.05	0.17	0.808	0.908	-1.23	0.19	0.258	0.648	**-1.45**	**0.06**	**0.000**	**0.003**
Slc12a5	**1.43**	**0.18**	**0.039**	**0.168**	1.03	0.17	0.882	0.946	-1.12	0.05	0.151	0.331
Slc17a6	-1.1	0.19	0.705	0.903	-1.11	0.17	0.634	0.811	-1.04	0.10	0.841	0.881
Slc17a7	**-6.00**	**1.01**	**0.003**	0.076	-1.77	1.89	0.786	0.940	-2.58	1.08	0.316	0.428
Tac1	-1.08	0.14	0.651	0.903	-1.28	0.19	0.236	0.648	-1.38	0.14	0.064	0.186
